# Computational design of class II MHC binding peptide with sequence-based evolution information

**DOI:** 10.1093/bioadv/vbag090

**Published:** 2026-05-08

**Authors:** Ying Cao, Yuqing Li, Weitong Ren, Wenfei Li, Zhiqiang Yan

**Affiliations:** Postgraduate Training Base Alliance, Wenzhou Medical University, Wenzhou, Zhejiang Province 325000, China; Zhejiang Key Laboratory of Soft Matter Biomedical Materials, Wenzhou Institute, University of Chinese Academy of Sciences, Wenzhou, Zhejiang Province 325000, China; Zhejiang Key Laboratory of Soft Matter Biomedical Materials, Wenzhou Institute, University of Chinese Academy of Sciences, Wenzhou, Zhejiang Province 325000, China; Zhejiang Key Laboratory of Petrochemical Environmental Pollution Control, National Engineering Research Center for Marine Aquaculture, Marine Science and Technology College, Zhoushan, Zhejiang Province 316004, China; Zhejiang Key Laboratory of Soft Matter Biomedical Materials, Wenzhou Institute, University of Chinese Academy of Sciences, Wenzhou, Zhejiang Province 325000, China; Zhejiang Key Laboratory of Soft Matter Biomedical Materials, Wenzhou Institute, University of Chinese Academy of Sciences, Wenzhou, Zhejiang Province 325000, China; School of Physics, National Laboratory of Solid State Microstructure, Nanjing University, Nanjing, Jiangsu Province 210093, China; Postgraduate Training Base Alliance, Wenzhou Medical University, Wenzhou, Zhejiang Province 325000, China; Zhejiang Key Laboratory of Soft Matter Biomedical Materials, Wenzhou Institute, University of Chinese Academy of Sciences, Wenzhou, Zhejiang Province 325000, China

## Abstract

MHCII-peptide binding plays a vital role in immunology. MHCII molecules are primarily expressed on the surface of antigen presenting cells where they capture and present exogenous antigen peptides to helper T cells, thereby activating humoral and cellular immune responses. Designing artificial MHCII binding peptides mimicking native peptides is crucial for vaccine development. However, it is challenge to design artificial binding peptides with conventional structure-based methods since the structures of the peptides are highly flexible at unbound state. In this work, we trained Transformer neural network to design the artificial peptides with sequence-based evolutionary information including the frequency distribution of amino acids at each site and the joint frequency distribution between amino acid pairs extracted from multiple sequence alignment of native peptides. In light of accurate sequence-based scoring function and reliable AlphaFold3 for complex structure prediction, the designed artificial peptides were predicted to have comparable binding affinities as native peptides and high structural confidence (pLDDT > 90.0) when binding to MHCII. Our work establishes a paradigm for designing different kinds of functional peptides and will greatly provide significant assistance to biomedical researchers in the medical and industrial fields.

## 1. Introduction

The interaction between MHC (Major Histocompatibility Complex) molecules and peptides is central to immune response (Janeway *et al*. 2001, Hartout *et al*. 2023). MHCII molecules present specific peptides derived from endosomal proteins for recognition by T helper cells. MHCII molecules are transmembrane glycoprotein heterodimers, which capture exogenous antigenic peptides through their peptide binding region. MHCII-binding peptides adopt a conserved binding mode, this canonical binding mode consists of a linear 9-mer binding core on the peptide sequence, and peptide-flanking residues extends on the N- and C-terminal parts of the binding core. The binding core contribute most of the interactions with the MHCII binding site. Therefore, designing peptides, especially the binding cores that bind to MHCII molecules is critical to understand the mechanisms of the immune response, identify T cell epitopes, and develop new vaccines and immunotherapies. Human MHCII molecules are encoded in the human leukocyte antigen (HLA) gene complex, and encompassing three major types of molecules: DP, DQ, and DR (Traherne 2008, Dubrot *et al*. 2014, Spanier *et al*. 2016). The high degree of polymorphism conferred by multiple allelic variants significantly increases the complexity of MHCII peptide binding prediction and de novo design (Chaffey *et al*. 2003, Blum *et al*. 2013, Paola Cruz-Tapias and Anaya 2013, Kuksa *et al*. 2015, Alvarez *et al*. 2018, Jurtz and Olsen 2018, Alspach *et al*. 2019, Kim *et al*. 2023). Over the past decade, extensive efforts have been devoted to developing computational methods for both identifying MHCII-binding peptides and predicting their binding affinities. With the advent of deep learning technology, significant progress has been made in the study of pan-specific MHC-peptide interactions (Zhang *et al*. 2012, Reynisson *et al*. 2020, You *et al*. 2022, Mukhopadhyay 2023). By training based on a wide range of MHC ligands and binding data, predictors such as NetMHCIIpan-4.1, MixMHC2pred and DeepMHCII, have achieved remarkably high prediction accuracy.

However, the design of MHCII-binding peptides still faces significant challenges, as neither traditional experimental approaches nor machine learning methods have achieved efficient design (Pineda-Castañeda *et al*. 2020, Shah *et al*. 2024). This dilemma primarily stems from the unique structural characteristics of MHCII-binding peptides. These peptide sequences are relatively short ranging from 13 to 25 amino acids in length and their binding cores being even shorter with typically 9 amino acids, this limited length prevents the peptides from forming stable structure. Given that the MHCII binding peptides lack stable secondary structures, they cannot be effectively designed using conventional or AI-based methods which rely heavily on the structural information (Madani *et al*. 2023, Yu and Buehler 2020, Ovchinnikov and Huang 2021, Dauparas *et al*. 2022, Ingraham *et al*. 2023).

Recently, there have been many successful reports demonstrating that proteins can be designed based entirely on sequence-based evolutionary information without relying on known structures (Socolich *et al*. 2005, Brender *et al*. 2017, Russ *et al*. 2020). Through the alignment and analysis of the amino acid sequences of homologous proteins, evolutionary information of homologous proteins can be effectively extracted. It has been observed that amino acid residues among protein family members exhibit significant evolutionary correlations, and this correlation mechanism plays a decisive role in maintaining the stable structure and specific interactions of proteins (Morcos *et al*. 2011, Marks *et al*. 2012, Ovchinnikov *et al*. 2017, Slater *et al*. 2020, Yan and Wang 2020, Baek *et al*. 2021, Pereira *et al*. 2021). For the target protein family, quantitative analysis of multiple sequence alignment (MSA) data using statistical modeling methods can capture key sequence features associated with function. At the single-residue level, conservation analysis identifies sites critical to protein function or structural stability. At the residue-pair level, the two-site co-evolution analysis captures the synergistic variation patterns across homologous sequences, thereby revealing the functional or structural coupling relationships between residues (Gueudré *et al*. 2016, Figliuzzi *et al*. 2018, Russ *et al*. 2020).

Short peptides are often disordered, but with access to large amounts of well-aligned peptide sequence data, one can leverage evolutionary information extracted from MSAs to a great extent (Socolich *et al*. 2005, Carbone and Dib 2011, Yan and Wang 2024). Unlike established prediction tools which assess the binding capability of given peptide-MHCII pairs (Racle *et al*. 2023), our method addresses the inverse design problem: de novo design of new binding peptide sequences targeting specific HLA allotype. Our method is uniquely grounded in sequence analysis, as short peptide ligands typically lack stable secondary structures, eliminating the need for structural information. In this study, we developed a deep learning Transformer framework that automatically learns conservative and coupled evolutionary patterns to generate artificial peptide sequences. By integrating evolutionary information with machine learning, this method aims to generate designed sequences which can not only retain key anchor residues but also maintain their intrinsic correlations. We also employed an alternative computational approach, i.e. the Monte Carlo simulated annealing algorithm, to cross-validate the artificial sequences generated by the Transformer neural network. Both algorithms donstrated that the designed short peptides not only retain the conservation of key functional sites but also maintain residue-residue coevolution. To validate the binding capacity of the designed peptides, we used the deep learning-based model DeepMHCII (You *et al*. 2022) to calculate their binding affinity with MHCII molecules and AlphaFold3 to predict the structure of MHCII-peptide complexes (Abramson *et al*. 2024). The results demonstrate that the designed short peptides not only exhibit high affinity for MHCII molecules but also form stable complexes with MHCII (and TCR/antigen) with high structural confidence. This finding confirms that the peptide sequences generated by our design strategy match native peptides in terms of binding patterns and structural characteristics, representing a significant advance in the rational design of short peptides.

## 2. Methods

### 2.1. Native sequence data of MHCII binding peptides

**Figure 1:**
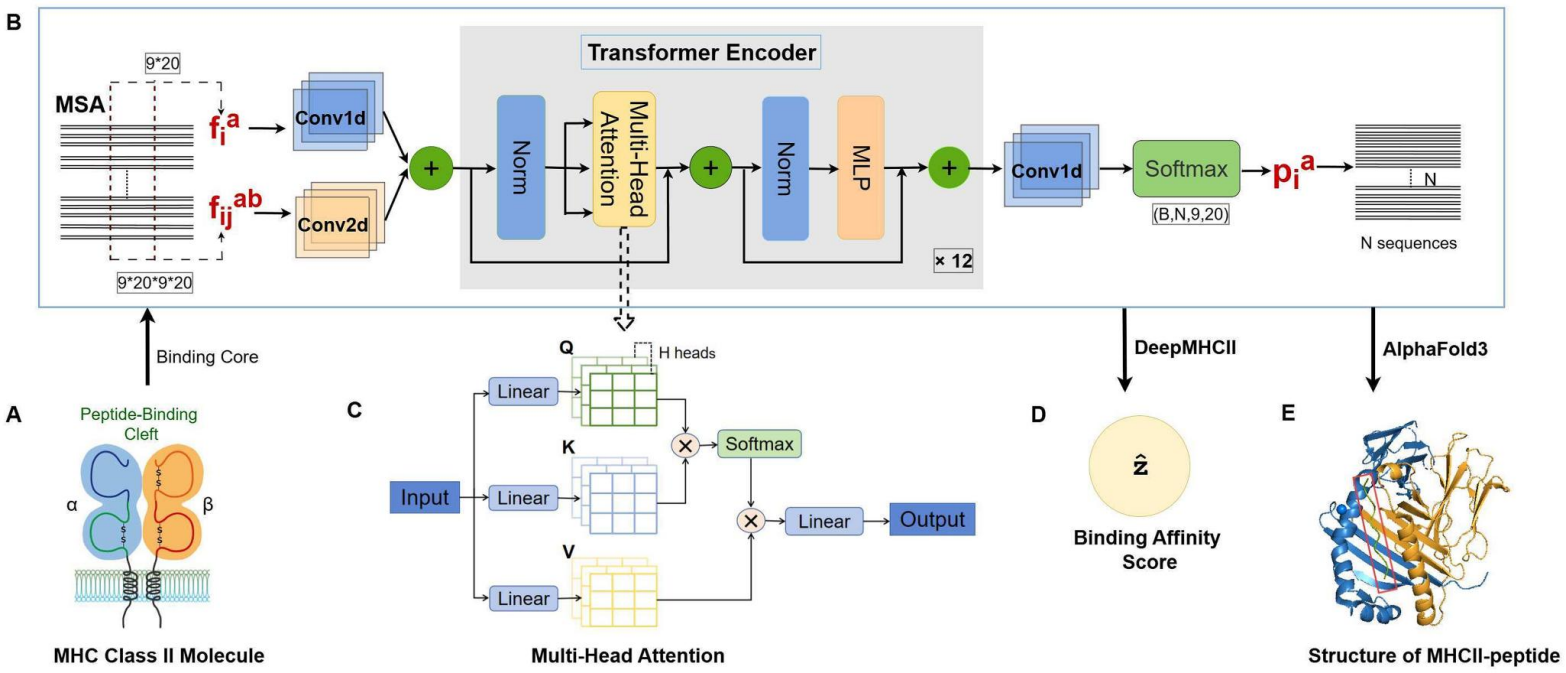
Flowchart of MHCII-peptide binding cores design using a Transformer neural network. (A) Schematic of the MHCII molecule, comprising α and β chains that form a peptide-binding cleft. (B) The neural network integrates convolutional and Transformer modules to learn sequence evolutionary features from MSA data for binding core design. (C) Flowchart of multi-head attention calculation: The input sequence is linearly projected into Query (Q), Key (K), and Value (V) matrices, each split into H attention heads. Each head independently computes attention weights; outputs are then concatenated and passed through a final linear projection. (D) DeepMHCII predicts the affinity score of the designed binding core with MHCII. (E) AlphaFold3 predicts the structure of the complex formed by the designed binding core and MHCII.

The peptide-binding region of MHCII is located at the interface between the α and β chains, with binding grooves open at both ends to accommodate peptides of varying lengths (Fig. 1A). The MHCII binding interface contains nine characteristic pockets that collectively form a peptide-binding cleft, where each pocket specifically recognizes one amino acid residue of the bound peptide (Janeway *et al*. 2001). These nine residues constitute the peptide’s binding core. In this study, we aim to design novel binding cores by analyzing the evolutionary information of native binding cores that bind to three classical human MHCII subtypes (HLA-DP, -DQ, and -DR).

Native MHCII-binding peptides were obtained from two independent datasets: (1) BD2020 (pre-2020 literature data): Extracted from IEDB by Vita *et al*. (2010) for training MHCAttnNet (Venkatesh *et al*. 2020). We screened samples to include only those with no fewer than 200 corresponding peptide sequences for each MHCII molecule. (2) Dset_pos_ (new data added post-2020): Downloaded from IEDB by Yang *et al*. (2024) for evaluating the performance of different predictor models. Similarly, each MHCII molecule was screened to include no fewer than 200 peptide sequences. Combining these two datasets, we obtained a total of 46,145 peptide sequences (42783 sequences remaining after filtering) that bind to 27 HLA allotypes (17 DR, 1 DQ, and 9 DP). Subsequently, we used NetMHCIIpan-4.1 (Reynisson *et al*. 2020) to precisely predict the binding core for each collected MHCII–peptide complex. We also used the latest NetMHCIIpan-4.3, we confirm that the binding core predictions are highly consistent between the two versions for randomly selected HLA-DRB4*01:03 (the results have been compiled in a Supplementary Excel file (Supplementary Dataset 1), available as supplementary data at *Bioinformatics Advances* online). The resulting binding core data, designated as Dset_bc, served as the native MSA sequences and the evolutionary information is extracted from the data to train the network. The distribution of binding cores for each HLA allotype is shown in Supplementary Table S1, available as supplementary data at *Bioinformatics Advances* online.

### 2.2. Evolution information extracted from MSA

#### 2.2.1. Amino acid frequency and position conservation

Amino acid frequencies refer to the statistical probability distribution of different amino acids at specific positions within a MSA of homologous proteins. By calculating the amino acid frequency distribution at each position, we can systematically identify highly conserved sites and those with high variability. Positional conservation is a quantitative measure of the degree to which amino acid sites remain invariant during protein evolution. Highly conserved positions typically correspond to critical residues that maintain protein structure or function, whereas highly variable positions are subject to lower structural or functional constraints. First-order conservation is computed using Kullback–Leibler divergence (or relative entropy) (Rivoire *et al*. 2016), defined as:


(1) $$C_{i}=\sum_{a=1}^{20}f_{i}^{a}\ln(f_{i}^{a}/q^{a})$$

Where $f_{i}^{a}$ represents the observed frequency of residue type a at position i (across the 20 canonical amino acids), and $q^{a}$ denotes the background frequency. The intensity of $C_{i}$ reflects the functional or structural importance of the positions.

#### 2.2.2. Joint frequency and coupling conservation

In a family of homologous proteins, the joint frequency of the amino acid pair (*a*, *b*) at positions *i* and *j* is defined as the fraction of sequences in which residue a occurs at position *i* and residue *b* occurs at position j simultaneously. This pairwise amino acid joint frequency reflects the covariation relationship between amino acid pairs at different positions within the MSA. Coupling conservation analysis can reveal coevolutionary patterns within peptides, indicating that these positions may be functionally or structurally related. To characterize pairwise amino acid coupling from MSAs, we used second-order conservation analysis to quantify coevolutionary patterns. The coupling strength between residue positions (*i*, *j*) is calculated as follows:


(2) $$C_{ij}=\sqrt{\sum_{a,b}{\left(k_{i}^{a}k_{j}^{b}\right)}^{2}{\left(f_{ij}^{ab}-f_{i}^{a}f_{j}^{b}\right)}^{2}}$$

Where $k_{i}^{a}=\ln[(f_{i}^{a}(1-q^{a}))/(q^{a}(1-f_{i}^{a}))]$, the $k_{i}^{a}$(or $k_{j}^{b}$) is the coefficient of the residue *a* (or residue *b*) position conservation function on position *i* (or position *j*). In MSA, $f_{ij}^{ab}$ represents the joint frequency of amino acid pairs type a appearing at position i and residue type b appearing at position j. Detailed derivations of these relationships can be found in Reference (Rivoire *et al*. 2016).

#### 2.2.3. Design artificial peptide sequences with evolution information

To design novel binding cores based on evolutionary information from Dset_bc, we implemented three distinct strategies:

1. Frequency-only based design: This strategy generates new sequences (designated as FS) by randomly sampling amino acids according to the amino acid frequency distribution at each site in native sequences, thereby retaining the first-order conservation patterns of native sequences.
2. Joint frequency-only or coupling-only design: This strategy leverages the pairwise amino acid coevolutionary relationships in Dset_bc. For the Transformer neural network, sequences are designed by learning features of pairwise amino acid joint frequencies. For the Monte Carlo simulated annealing algorithm, sequences are designed by optimizing the coupling function.
3. Integrated frequency and joint frequency/coupling design: This optimization strategy integrates site-specific amino acid frequencies with either pairwise amino acid joint frequencies or coupling conservation from Dset_bc. For the Transformer neural network, sequences are designed by learning features of both amino acid frequencies and pairwise amino acid joint frequencies. For the Monte Carlo simulated annealing algorithm, sequences are designed by optimizing coupling values of amino acid pairs while maintaining single-amino-acid frequency constraints.

The neural network and Monte Carlo methods mutually validate each other. Ultimately, five types of designs for peptide sequences based on evolutionary information were generated, with details described below.

#### 2.2.4. Training with neural network of transformer

##### 2.2.4.1. Input and convolutional layer

The binding cores specific to individual MHCII-peptide complexes are represented with a rectangular matrix $a=\{a_{\mu i}|i=1,\ldots ,L,\mu =1,\ldots ,M\}$, containing *M* sequences, which are aligned at *L* positions (*L* = 9). For each HLA allotype-binding cores, we calculated both amino acid frequency and pairwise amino acid joint frequency, which form two-dimensional tensors and four-dimensional tensors respectively. The positional amino acid frequency tensors $F\in R^{9*20}$ (flattened to $R^{1*180}$) was processed using 1D convolutional neural network (CNN) to obtain a high dimension matrix $F^{\prime }$ for extracting local amino acid frequency features. The pairwise amino acid joint frequency tensors $T\in R^{9*20*9*20}$ (reshaped to $R^{180*180}$) was analyzed via 2D CNN to obtain a high dimension matrix $T^{\prime }$ for capturing local joint frequency feature. These two types of convolved features were subsequently concatenated along the feature dimension to implement feature fusion, that was:


(3) $$Z=\text{Concat}(F^{\prime },T^{\prime })$$

The fused feature Z was then fed into the Transformer module (Fig. 1B). This procedure was applied to the binding cores of all 27 HLA allotypes, which collectively constitute the input for training the neural network.

#### 2.2.5. Transformer

In this module, the processed representations of amino acid frequencies and pairwise amino acid joint frequencies were fed into a stack of 12 Transformer blocks, enabling the extraction of high-order frequency dependencies to capture complex patterns within the sequences (Han *et al*. 2021). Each Transformer block was centered on the Multi-Head Self-Attention (MHSA) mechanism, which projected input features into three distinct spaces via independent linear transformations, generating Query (*Q*), Key (*K*), and Value (*V*) matrices (Fig. 1C). These representations were then split into H parallel attention heads, with each head dynamically attending to different positions of the input sequence through scaled dot-product attention. The formula for calculating attention weights is as follows:


(4) $$\text{Attention}(Q_{i},K_{i})=\text{Softmax}\left(\frac{Q_{i}K_{i}^{T}}{\sqrt{d_{k}}}\right)$$

Where $Q_{i}$ and $K_{i}$ denote the query and key matrices for the *i*-th attention head, respectively, each obtained from the input sequence via learned linear projections. $K_{i}^{T}$is the transpose of the key matrix, $d_{k}$ represents the dimensionality of the key vectors. The attention-weighted Value outputs from all heads were concatenated and passed through a linear projection to form the final representation. By leveraging multi-head attention, the Transformer architecture captures global dependencies between any residue pairs, overcoming the local receptive field limitations of conventional CNNs and RNNs.

#### 2.2.6. Output layer

The globally extracted features from the Transformer were further processed through 1D convolutional layers to project them into task-specific outputs, followed by a Softmax activation function to ensure probabilistic outputs with smooth gradients for more stable training (Fig. 1B). For each sequence, the model outputs the probability $P_{i}^{a}$ of the 20 amino acids (denoted as *a*) at each position *i* (*i* = 1,2,…9). Candidate peptide sequences were then generated by sampling amino acids according to these position-specific probability distributions. The details of the process for generating the final sequences were displayed in Supplementary Methods 1.1, available as supplementary data at *Bioinformatics Advances* online.

#### 2.2.7. Loss function

In this study, two loss functions were designed to guide peptide sequence generation. The first uses pairwise amino acid joint frequency information from Dset_bc as input; here, the model employs mean square error (MSE) between the model-predicted joint frequencies and the native joint frequencies as the loss function. The second uses both amino acid frequencies and pairwise amino acid joint frequencies from Dset_bc as input, with MSE between the predicted amino acid frequencies, predicted joint frequencies and those from native sequences as the loss function. When only pairwise joint frequencies were used as the loss of the network, the formula was as follows:


(5) $$\begin{array}{c} \text{Loss}1=\text{MSE}\left(f_{ij}^{ab},{\hat{f}}_{ij}^{ab}\right)\\ =\frac{1}{B}\sum_{n=1}^{B}\frac{1}{{\left(9\times 20\right)}^{2}}\sum_{i,j=1}^{9}\sum_{a,b=1}^{20}{\left(f_{n,i,j}^{a,b}-{\hat{f}}_{n,i,j}^{a,b}\right)}^{2} \end{array}$$

Where ${\hat{f}}_{ij}^{ab}$ denotes the joint frequency of residue type *a* at position *i* and residue type *b* at position *j* in native sequences, and B is the batch size. $f_{n,i,j}^{a,b}$, ${\hat{f}}_{ij}^{ab}$ respectively represents the predicted frequencies and native joint frequencies of amino acids at positions i and j in the binding cores of peptide sequences corresponding to the *n*-th MHCII allele. When both first-order and second-order evolutionary information were used as the loss of the network, the calculation formula was as follows:


(6) $$\begin{array}{l} \text{Loss}2=\text{MSE}(f_{i}^{a},{\hat{f}}_{i}^{a})+\lambda \text{Loss}1=\frac{1}{B}\sum_{n=1}^{B}\sum_{i=1}^{9}\sum_{a=1}^{20}{\left(f_{n,i}^{a}-{\hat{f}}_{n,i}^{a}\right)}^{2}\\ +\frac{\lambda }{B{\left(9\times 20\right)}^{2}}\sum_{n=1}^{B}\sum_{i,j=1}^{9}\sum_{a,b=1}^{20}{\left(f_{n,i,j}^{a,b}-{\hat{f}}_{n,i,j}^{a,b}\right)}^{2} \end{array}$$

Where λ is a coefficient, which was set to λ = 65 to balance the contribution of the two loss terms (The selection of λ and a sensitivity analysis were explained in the Supplementary Methods 1.2, available as supplementary data at *Bioinformatics Advances* online). ${\hat{f}}_{i}^{a}$ denotes the frequency of residue type a in the native sequence at position i. $f_{n,i}^{a}$, ${\hat{f}}_{n,i}^{a}$ respectively represent the predicted frequencies and native frequencies of amino acid a at position i in the binding cores of peptide sequences corresponding to the n-th MHCII allele. The changes in both loss terms are shown in Supplementary Fig. S1, available as supplementary data at *Bioinformatics Advances* online.

During the training process, the Adam optimizer (with a learning rate 1e-4) was employed for 2000 training epochs, and network parameters were updated via backpropagation by monitoring the descending trend of the loss function. Training was terminated when the loss value converged (Supplementary Fig. S1, available as supplementary data at *Bioinformatics Advances* online). Designed sequences generated using Loss1 and Loss2 were designated as L1 and L2 sequences, respectively. L1 sequences are intended to recapitulate the amino acid pairwise joint frequencies of native peptides, while L2 sequences are designed to simultaneously retain both amino acid frequency and pairwise amino acid joint frequency features. To ensure robustness, the model was trained independently ten times to verify whether all runs can produce highly consistent outputs.

#### 2.2.8. Design peptide sequences with Monte Carlo Simulation Annealing

##### 2.2.8.1. Inputs and optimization

In protein sequence design, the Monte Carlo Simulated Annealing (MCSA) algorithm effectively explores the vast sequence space and optimizes an objective function to generate protein sequences with desired statistical properties. Unlike the neural network model which used mixed inputs, this algorithm selects binding cores specific to individual HLA class II allotypes (e.g., HLA-DRB5*01:01), represented as a matrix $a=\{a_{\mu i}|i=1,\ldots ,L,\mu =1,\ldots ,M\}$, containing M sequences, as input data for each run. The algorithm employs an iterative architecture with outer and inner loops. The outer loop controls temperature, consisting of 2000 annealing cycles. The initial temperature was set to 15, and each annealing step followed the exponential decay law $T_{k+1}=0.99T_{k}$, where $T_{k+1}$ is the temperature parameter for the next round and $T_{k}$ is the current temperature parameter. The inner loop involves 2000 iterations of MCSA sequence space search at each constant temperature. In each iteration, amino acids at each position of the sequences were mutated with a 8% probability across all M sequences to explore the optimal solution of the objective function at that temperature. The parameters selection process and parameters sensitivity analysis were described in detail in the Supplementary Methods 1.3, available as supplementary data at *Bioinformatics Advances* online.

This study calculated the amino acid frequency distribution at each position to assess the first-order conservation characteristics of the sequences, and employed second-order (coupling) conservation analysis to quantitatively characterize the co-evolutionary relationships between amino acid pairs. Based on these evolutionary statistics, we developed two distinct sequence design strategies: coupling-only, and integrated frequency and coupling. Both strategies utilized the square root of the differences in coupling matrices between native and designed sequences as the objective function in the MCSA algorithm:


(7) $$E=\sqrt{\sum_{i,j}{\left(C_{ij}^{\text{Design}}-C_{ij}^{\text{Native}}\right)}^{2}}$$

The diagonal elements of the coupling matrix exhibit strong conservation and dominate the objective function. To improve stability, their weight was reduced to 0.1, allowing the optimization to focus on off-diagonal coupling variations.

These two strategies generate MC1 and MC2 sequences, respectively. Notably, MC1 sequences employ completely randomized initialization and mutation processes. MC2 sequences, by contrast, involve both initialization with FS sequences and mutations guided by the position-specific amino acid frequencies of native sequences, enabling synergistic optimization of first-order and second-order conservation. Whether a mutated sequence is retained depends on the objective function: if the objective function value of the new solution is smaller (i.e., the difference is negative), the solution is accepted directly. Otherwise, the inferior solution is accepted with a probability of $\text{exp}\;\left(-\frac{\Delta E}{T}\right)$ according to the Metropolis criterion, thereby avoiding convergence to a local optimum.

#### 2.2.9. Output

As the number of iterations increases, the algorithm rejects new solutions over several consecutive attempts (Supplementary Fig. S2A, available as supplementary data at *Bioinformatics Advances* online). Meanwhile, the objective function shows a steady downward trend and eventually converges (Supplementary Fig. S2B, available as supplementary data at *Bioinformatics Advances* online). At the completion of the algorithm, a set of optimized MHCII-peptide-specific binding cores are generated. Through iterative optimization, MCSA calculates the coupling strength between amino acid pairs, enabling artificially designed peptide sequences to recapitulate the observed coupling patterns in native peptide sequences.

#### 2.2.10. Validation of designed sequences

##### 2.2.10.1. Binding affinity prediction with DeepMHCII

In the field of MHCII-peptide interaction prediction, deep learning-based methods have demonstrated superior accuracy over traditional approaches (Zhang *et al*. 2012, Reynisson *et al*. 2020, You *et al*. 2022, Mukhopadhyay 2023). Because binding cores are relatively short and typically exhibit weaker affinity than full-length peptides, we rigorously selected high-affinity binding cores (NetMHCIIpan-4.1-predicted IC50 < 50 nM) from the Dset_pos_ and BD2020 datasets to ensure a pronounced affinity contrast between randomly generated and native peptides. These native high-affinity binding cores were then used to design peptides (FS, L1, L2, MC1, MC2).

The core-aware deep interaction model, named DeepMHCII, was employed in this study to predict MHCII-peptide binding affinity and validate the artificially designed peptides. We trained 100 sub-models using 5-fold cross-validation over 20 epochs. During testing, both the designed peptides and their corresponding HLA allotypes were input into the DeepMHCII model. The model performed forward propagation across all 100 trained sub-models, with the average prediction used as the final binding affinity score áº‘ (Fig. 1D). DeepMHCII is publicly available at https://github.com/yourh/DeepMHCII. We further employed NetMHCIIpan-4.3 (Nilsson *et al*. 2023) to predict the binding affinity of our designed peptides, and the details were shown in Supplementary Methods 1.4, available as supplementary data at *Bioinformatics Advances* online.

#### 2.2.11. Structural prediction with AlphaFold3

AlphaFold3 (Abramson *et al*. 2024) has achieved groundbreaking progress in predicting biomolecular complex structures, enabling accurate modeling of both single proteins and their complexes with other biomolecules (e.g., DNA, RNA, ligands). We submitted the designed binding core sequences, along with their corresponding MHCII molecules and either T-cell receptors (TCRs) or potential interacting antigens, to AlphaFold3 for structural prediction. AlphaFold3 enables the direct acquisition of high-precision structural information without the need for extensive experimental procedures (Fig. 1E), effectively reducing the time and resource demands associated with traditional methods.

## 3. Results

Peptide sequences capable of binding to 27 HLA allotypes were tested to design their corresponding binding cores. Among these MHCII molecules, the HLA-DRB5*01:01 allele was selected as an illustrative example because it has a resolved PDB structure with its binding peptide and interacting antigens (PDB ID: 1HQR), facilitating subsequent comparisons. In this section, we focus on designing binding cores that bind to the HLA-DRB5*01:01 allele, highlighting results for binding cores designed using the Transformer neural network method based on evolutionary information. Testing results for binding cores targeting the HLA-DRB5*01:01 allele—designed using Monte Carlo simulated annealing with evolutionary information—are presented in the Supplementary Materials (Supplementary Figs. S3 and S4 and S5, available as supplementary data at *Bioinformatics Advances* online). Additionally, to ensure the robustness of the computational strategies, testing results for Transformer neural network–designed binding cores targeting HLA-DPA1*01:03_DPB1*02:01 are provided in the Supplementary Materials (Supplementary Figs. S6 and S7, available as supplementary data at *Bioinformatics Advances* online).

### 3.1. Evolutionary information of designed peptide sequences

**Figure 2:**
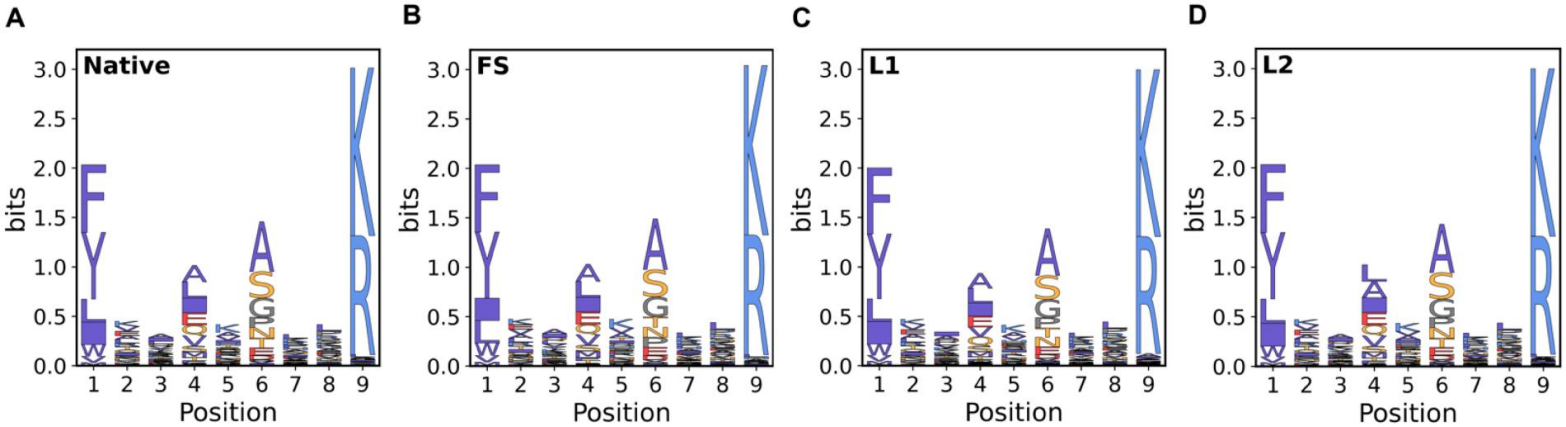
First-order conservation profiles of 9-mer binding cores for the HLA-DRB5*01:01 allele. (A) Native sequences. (B) FS sequences. (C) L1 sequences. (D) L2 sequences. The overall height of letters indicates the amino acid conservation at that position, while the height of each amino acid represents the relative frequency at that position.

**Figure 3:**
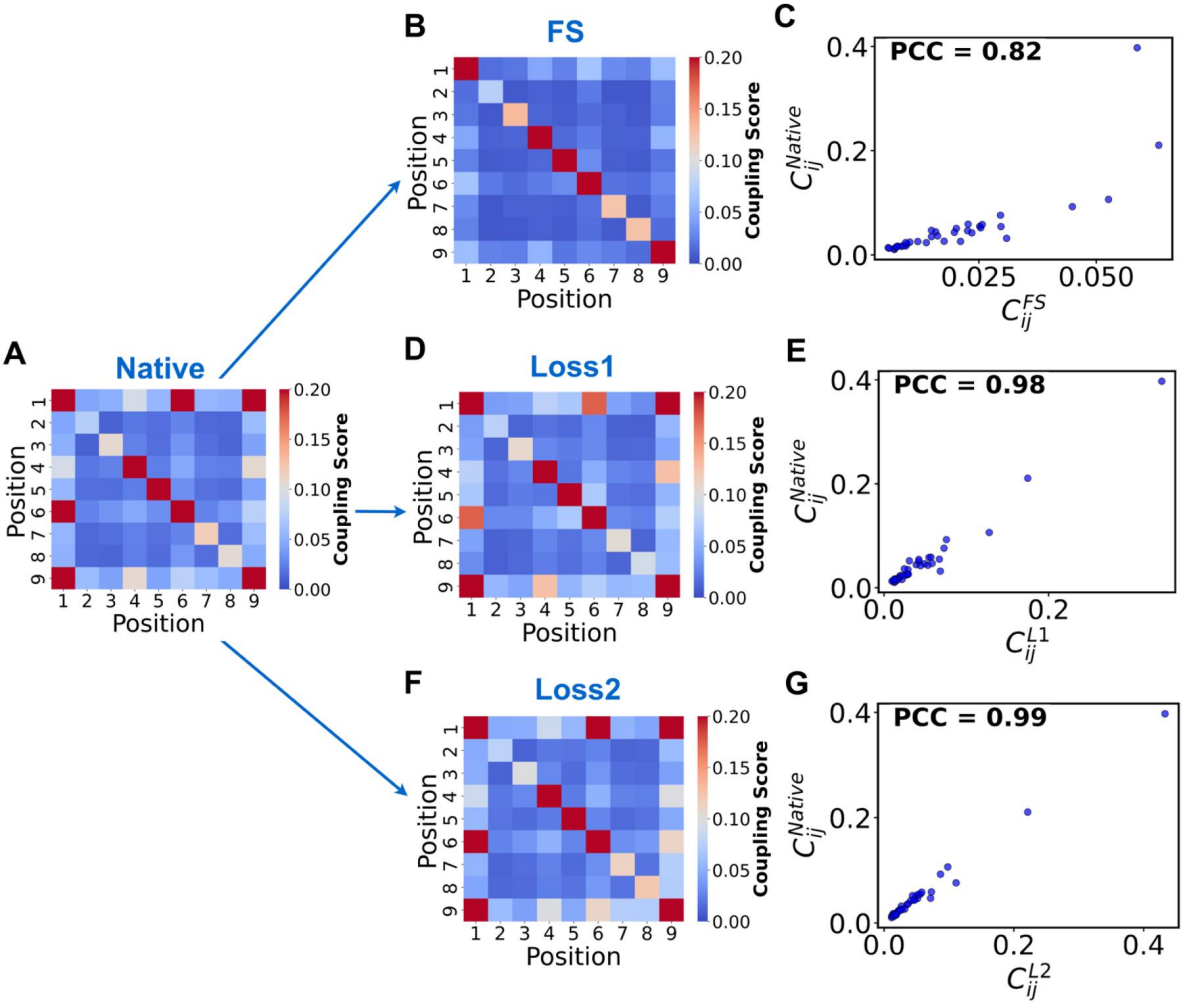
Second-order conservation analysis comparing designed 9-mer peptide binding cores for the HLA-DRB5*01:01 allele with native 9-mer binding cores across different design strategies. (A) Coupling matrix of native binding cores. (B) Coupling matrix of FS sequences. (C) Pearson correlation coefficient (PCC) of coupling scores between FS sequences and native sequences (0.82). (D) Coupling matrix of L1 sequences. (E) PCC of coupling scores between L1 sequences and native sequences (0.98). (F) Coupling matrix of L2 sequences. (G) PCC of coupling scores between L2 sequences and native sequences (0.99).

In this study, the native binding cores of HLA-DRB5*01:01-peptide complexes served as the benchmark dataset. (Fig. 2 and Supplementary Fig. S8, available as supplementary data at *Bioinformatics Advances* online) shows examples of conservation logos for peptides binding to HLA-DRB5*01:01. Plots for another allele, HLA-DPA1*01:03_DPB1*02:01, are available in Supplementary Fig. S9, available as supplementary data at *Bioinformatics Advances* online. In addition, We also supplemented conservation logos for the other three HLA allotypes in Supplementary Fig. S10, available as supplementary data at *Bioinformatics Advances* online. We observe that the first-order conservation distribution of binding cores designed using different strategies for HLA-DRB5*01:01 is highly consistent with that of native sequences. Positions (pockets) 1, 4, 6, and 9 all exhibit high conservation and are identified as key functional sites (Supplementary Table S2, available as supplementary data at *Bioinformatics Advances* online); this aligns with the consensus that P1 (pocket 1), P4, P6, and P9 are the four main anchor points (Rammensee *et al*. 1999, Crooks *et al*. 2004, You *et al*. 2022, Yang *et al*. 2024). Native sequences exhibit highly conserved sites and second-order conservation (Figs. 2A and 3A). Pearson correlation coefficient (PCC) analysis demonstrates that FS sequences maintain first-order conservation but lack significant second-order conservation (Figs 2B, 3B and C). In contrast, L1 and L2 sequences preserve both first-order and second-order conservation (Figs. 2 and 3). Specifically, the coupling matrices of L2 sequences and native sequences are highly similar. In addition, we have extended our analysis to include all 27 alleles present in the dataset. we conducted 10 independent training runs, calculating the PCC for each category of data from the resulting models each time, then took the average (Supplementary Table S3, available as supplementary data at *Bioinformatics Advances* online) and we computed the mean and standard deviation of the PCC values from five measurements within a single training run (Supplementary Table S4, available as supplementary data at *Bioinformatics Advances* online). The high scores of PCC for these different MHCII-binding cores designed by our network confirms that the neural network can learn the evolutionary features of native sequences.

### 3.2. Binding affinities of designed peptides to MHCII

**Figure 4:**
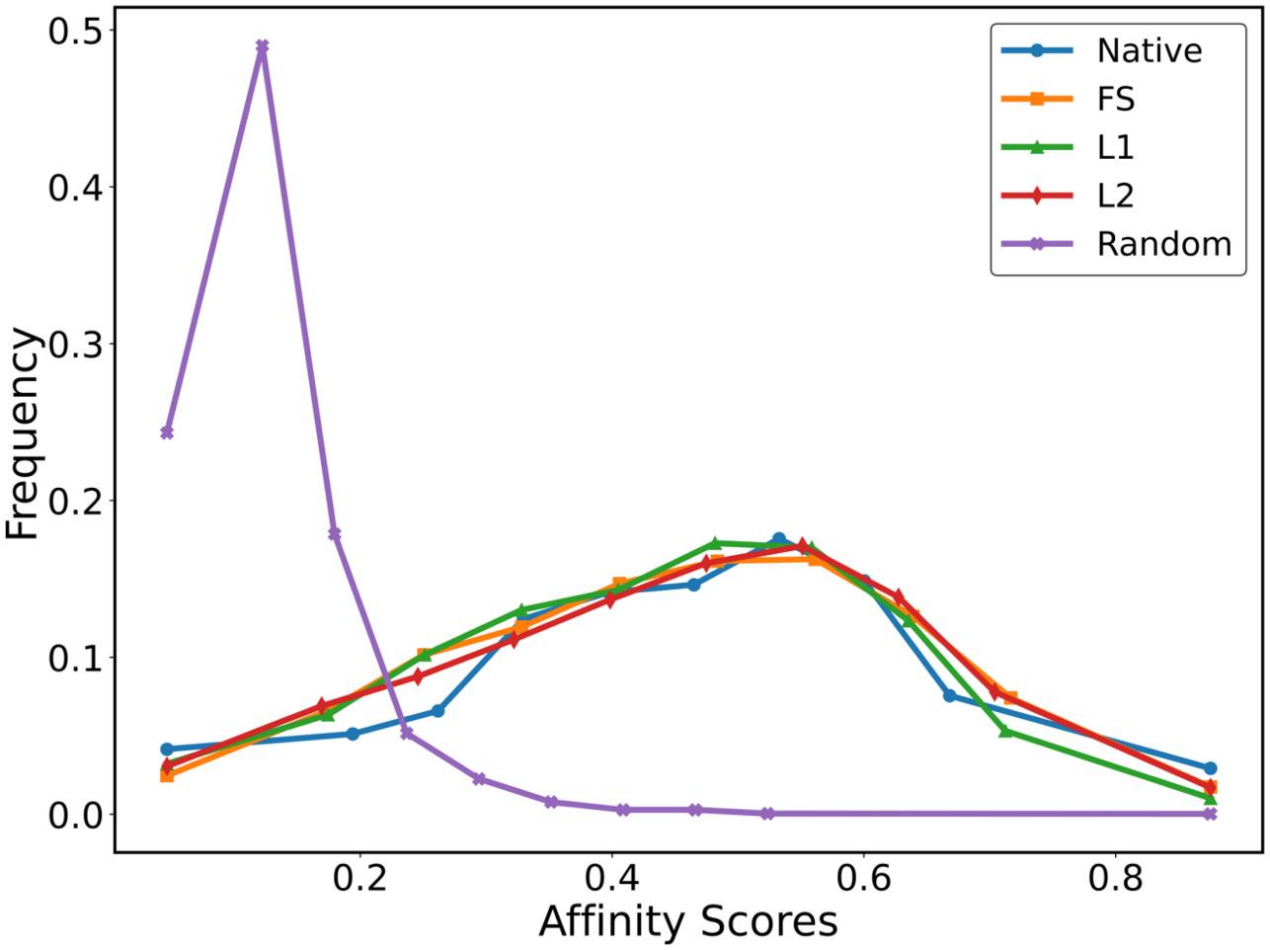
Binding affinity distributions of designed sequences, native sequences, and random sequences for the HLA-DRB5*01:01 allele.

This study employed the deep learning model DeepMHCII to predict MHCII-peptide binding affinity, comparing the binding affinities of designed peptides for MHCII with those of randomly generated sequences. Evolution-informed designed peptides exhibit binding affinities to the MHCII molecule that are similar to those of native MHCII-peptide complexes, whereas random peptides show significantly lower predicted affinity. Notably, L2 sequences exhibited superior predicted binding affinity distributions for the HLA-DRB5*01:01 allele compared to other types of designed sequences (Fig. 4), while maintaining consistently high affinity across different λ values and showing a strong correlation with natural sequences (Supplementary Table S5, available as supplementary data at *Bioinformatics Advances* online). Moreover, predicted binding affinity distributions generated by NetMHCIIpan-4.3 (Supplementary Fig. S11, available as supplementary data at *Bioinformatics Advances* online) also indicate that our designed sequences and the natural binding sequences exhibit similar patterns. These findings not only validate the theoretical hypothesis of synergistic optimization through amino acid frequency and pairwise joint frequency analysis but also demonstrate the feasibility of using pairwise amino acid joint frequency (coupling conservation) to develop MHCII-binding peptides.

To ensure result reliability, five independent L1 sequence sets and five independent L2 sequence sets were designed using the same trained model for reproducibility testing. High consistency in affinity distributions was observed across all replicates. (Supplementary Fig. S12, available as supplementary data at *Bioinformatics Advances* online), demonstrating that stochastic interference is effectively eliminated. Moreover, to ensure robustness, the model was trained independently 10 times, and all runs yielded highly consistent results (Supplementary Fig. S13, available as supplementary data at *Bioinformatics Advances* online). These results validate the stability of our design framework and establish a reliable basis for subsequent investigations.

Complex Structures of Designed Peptides Bound to MHCII and TCR/Superantigen

### 3.3. Comparison of pLDDT of peptides

**Figure 5:**
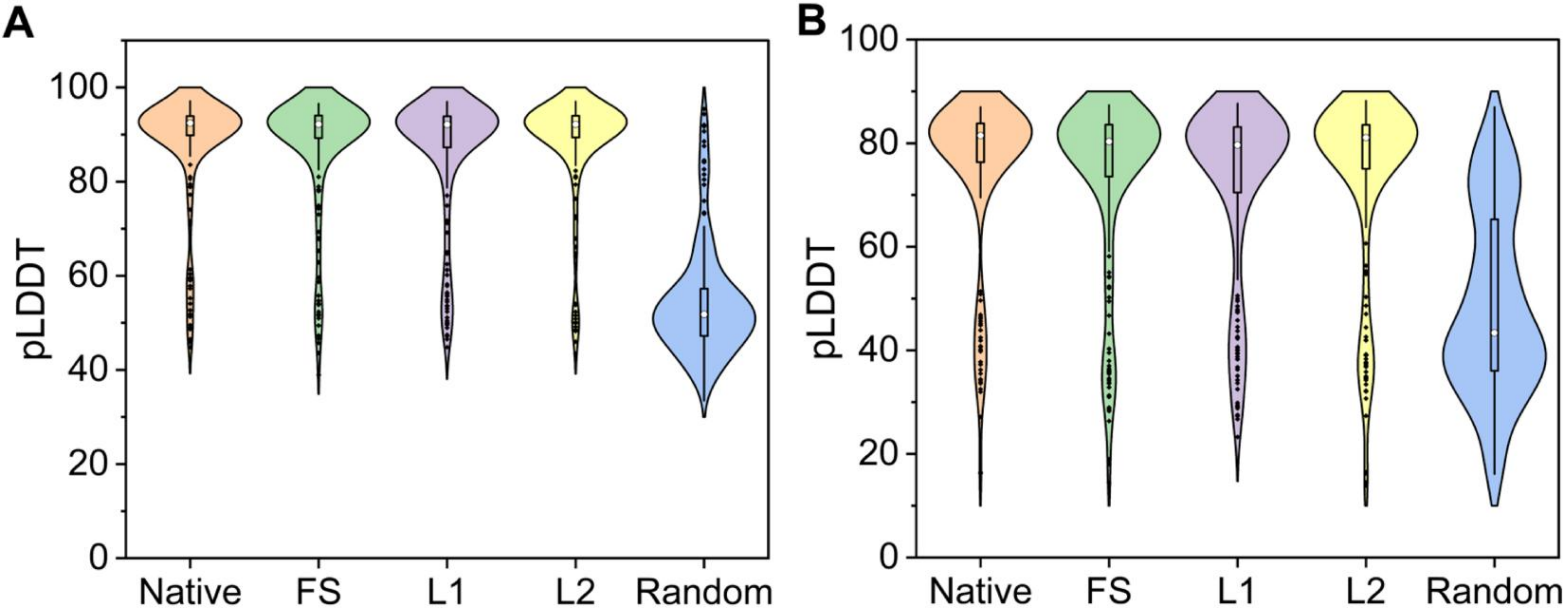
Distribution of pLDDT scores for five peptide groups binding the HLA-DRB5*01:01 allele, with 200 sequences sampled from each group. (A) For MHCII-peptide complexes: The average pLDDT scores of binding cores designed by different strategies are >90. (B) For MHCII-peptide-SPE-C complexes: The average pLDDT scores of binding cores designed by different strategies are >80.

The structural reliability of MHCII-peptide complexes was evaluated using AlphaFold3’s confidence metric, pLDDT (Predicted Local Distance Difference Test). The pLDDT score quantifies structural confidence through atomic-level and pairwise error estimation, with higher values indicating greater model reliability. From each peptide category (native, FS, L1, L2, random), 200 binding cores were randomly selected for structural prediction with HLA-DRB5*01:01. For each individual binding core, its pLDDT score was computed as the mean of all predicted atomic confidence values within that sequence. Results show that the pLDDT scores of designed peptides are comparable to those of native peptides and significantly higher than those of random peptides. Notably, L2 sequences achieved the highest pLDDT scores (indicating superior structural confidence) and the smallest fluctuation ranges (Fig. 5A). This large-scale validation confirms the structural reliability of the designed peptides.

**Figure 6:**
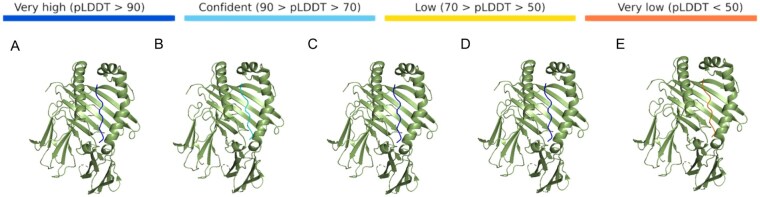
Confidence levels of binding to the HLA-DRB5*01:01 allele for binding cores designed using different strategies and randomized design. (A) Native sequences. (B) FS sequences. (C) L1 sequences. (D) L2 sequences. (E) Random sequences.

To further validate the robustness of our findings, one binding core was randomly selected from each of the five peptide categories (native, FS, L1, L2, random) for structural prediction with HLA-DRB5*01:01 using the AlphaFold3 server. A bluer color bar indicates a higher confidence level, revealing that designed peptides achieve higher confidence levels than randomly generated sequences (Fig. 6). This confirms the effectiveness of our design strategies. Notably, incorporating pairwise amino acid joint frequency in addition to amino acid frequency does not significantly improve pLDDT scores, suggesting limited coupling effects in short peptides. This finding provides critical guidance for future optimization of longer protein sequence designs (We further employed AlphaFold3 to predict the TM-score (iPTM) of the peptides, and the results revealed that their iPTM performance was consistent with that of pLDDT (Details in Supplementary Results and Supplementary Table S6, available as supplementary data at *Bioinformatics Advances* online)).

### 3.4. MHCII-peptide-SPE-C complex structure predicted by AlphaFold3

This study focuses on designing peptides that can bind to MHCII molecules and potentially modulate interactions involving bacterial superantigens (SAGs). We use PDB entry 1HQR (the crystal structure of a complex comprising the streptococcal superantigen SPE-C, the MHCII molecule HLA-DRB5*01:01, and a self-peptide derived from myelin basic protein (MBP)) as our research template. The literature (Li *et al*. 2001) indicates that the MBP-derived self-peptide not only binds to MHCII but also forms extensive contacts with SPE-C. This interaction mode, which resembles the peptide–MHCII-TCR interface, may regulate and enhance T-cell responses. Accordingly, the peptides designed in this study are expected to bind simultaneously to MHCII and SPE-C, fulfilling dual functional roles.

To evaluate the structural performance of our peptide designs, we replaced the native peptide sequence in the MHCII-peptide-SPE-C complex with the designed peptide sequences. From each peptide category (native, FS, L1, L2, random), 200 binding cores that bind to HLA-DRB5*01:01 were randomly selected, and their ternary complex structures with MHCII and the SPE-C superantigen were predicted using AlphaFold3. The average pLDDT scores of the peptides within these predicted complexes were calculated to assess structural confidence. Results show that designed peptides exhibit significantly higher pLDDT scores than random peptides (Fig. 5B). Notably, structural modeling reveals that the designed peptides maintain high-confidence conformations at the interface with both MHCII and SPE-C, closely resembling the native complex (Supplementary Fig. S14, available as supplementary data at *Bioinformatics Advances* online). The complex structures of designed peptides with another allele (HLA-DPA1*01:03_DPB1*02:01) and TCR are shown in Supplementary Fig. S15, available as supplementary data at *Bioinformatics Advances* online.

## 4. Discussion

In our computations, multiple strategies were employed to design MHCII-binding peptides. We designed these peptides by analyzing amino acid frequencies at each position alongside their co-evolutionary relationships (joint frequency or coupling conservation), followed by training with a Transformer neural network and optimization via Monte Carlo simulated annealing. Peptides designed using these strategies, except for MC1 sequences, nearly matched the performance of native peptide sequences. MC1 sequences only consider optimization of coupling functions without incorporating amino acid frequency constraints derived from native sequences; the poor structural confidence for the designed MC1 sequences indicates that frequency is an essential constraint in MCSA. Additionally, the MCSA method requires separate design of binding cores for each HLA allotype, rendering the approach computationally expensive. Notably, although MC2 sequences designed via MCSA are highly similar to those generated by the neural network method, the neural network method offers more significant advantages: it does not require explicit frequency constraints yet ensures reliable design outcomes. Moreover, the neural network exhibits superior computational efficiency, enabling it to effectively handle diverse MHCII–peptide binding core design tasks. This comparative study provides strong empirical support for the application of deep learning in protein design.

This study underscores the critical role of evolutionary information in peptide sequence design. Specifically, incorporating both amino acid frequencies and pairwise joint frequencies contributes to generating more stable and functionally effective peptide sequences. However, the improvement was not particularly pronounced, which may be attributed to the relatively short length of MHCII–peptide binding cores, limiting the evolutionary information that neural networks can extract. Despite this limitation, the findings provide valuable insights into peptide design and suggest that extending the approach to longer peptide sequences could further enhance its effectiveness. Future work could adapt these strategies to improve generalizability and broaden applicability for other peptide-design systems.

For the training data preparation, we employed NetMHCIIpan-4.1 to predict binding cores as references for natural peptide sequences, primarily due to the limited number of structurally resolved MHC-II-peptide complexes currently available in the PDB. NetMHCIIpan-4.1 is a widely validated core prediction tool trained on experimentally eluted ligand data. Given the current scarcity of experimental structural data, the use of such computational tools represents a necessary practice in our work. We acknowledge that the reliance on merely computational predictions of binding cores and binding affinities could constitute a limitation of this work, constrained by the scarcity of experimentally measured binding core data. However, this study primarily focuses on exploring properties of designed peptide sequences mimicking natural peptides, justifying two novel computational peptide design methods based on evolutionary information.

In addition, we acknowledge that there are overlaps between our training data and those of DeepMHCII. To exclude the potential influence of this factor on our validation results, we conducted an additional control implementation. Specifically, we removed all data points overlapping with the DeepMHCII training set and retrained our Transformer network using only the remaining non-overlapping data. The binding affinities of the novel peptides designed by our model were then also predicted by DeepMHCII. The results show that these newly designed peptides still exhibit an affinity distribution similar to that of naturally peptide sequences (as shown in Fig. S16). This observation indicates that by excluding the overlapping data, the design capability of our model are still validated by DeepMHCII. Moreover, it is worth emphasizing that in addition to binding affinity validation through DeepMHCII, the designed binding cores for given HLA allotypes were also validated in predicted MHCII-peptide structures through AlphaFold3, and in the evolutionary information similar as native peptides. Although this additional validation provides further support for the effectiveness of our model, we acknowledge that, as with any purely computational study, certain inherent limitations remain. In particular, the potential issue of predictive circularity, while mitigated here through the use of non-overlapping control data, cannot be entirely eliminated without experimental validation. Therefore, although our computational implementations—including independent training runs, external tool validation, and the overlap-removed control implementation—provide strong theoretical support for the biological plausibility of the designed peptides within an in silico framework, our model ultimately requires wet experimental validations.

## 5. Conclusion

We propose five strategies that leverage evolutionary information to design binding cores of MHCII-peptides using either neural networks or Monte Carlo simulated annealing. The designed binding cores exhibit near-native binding affinity to MHCII molecules and are accurately positioned within the peptide-binding cleft formed by both MHCII chains, with high structural confidence. Furthermore, structural modeling demonstrates that the designed peptides can form ternary complexes with MHCII and either superantigens or TCRs as native structures. Through extensive testings, we show that first-order and second-order sequence conservation are critical and play a key role in peptide sequence design. Incorporating pairwise amino acid joint frequency (coupling conservation) in addition to amino acid frequency distributions further enhances design accuracy and stability. By designing peptide sequences based on evolutionary information, researchers can more precisely regulate immune responses and develop more effective therapeutic and preventive strategies.

## Supplementary Material

vbag090_Supplementary_Data

## Data Availability

The customized data and code is publicly available at https://github.com/caoying2024-hue/peptide.
