# Supplementary material for: Computational design of class II MHC binding peptide with sequence-based evolution information
Source: Bioinform Adv. 2026 May 8;6(1):vbag090. doi: 10.1093/bioadv/vbag090 (PMC13154412; doi:10.1093/bioadv/vbag090)
Supplement: vbag090_Supplementary_Data [file vbag090_supplementary_data.zip › Supplementary_Materials.pdf]

Supplementary Materials for

Computational Design of Class II MHC Binding Peptide with Sequence-Based Evolution Information

Ying Cao, Yuqing Li ,Weitong Ren*, Wenfei Li*, Zhiqiang Yan*
*Email: weitong.ren@ucas.ac.cn, wfli@nju.edu.cn, zqyan@ucas.ac.cn

1. Supplementary Methods

1.1 Probability Distribution and Sampling Strategy.

The Transformer model outputs a probability distribution over the 20 standard amino acids for each of the 9 positions in the sequence of the binding core. This results in a probability tensor $P \in R^{N\times 9\times 20}$, where N is the total number of generated sequences, and P_i^a denotes the probability of amino acid a at position i. To generate concrete amino acid sequences, we employed multinomial sampling. For each position i in the 9-mer peptide, the corresponding 20-dimensional probability vector P_i was treated as the parameters of a multinomial distribution. Sampling from this discrete distribution allows for the selection of even low-probability amino acids. Unlike greedy sampling (which always selects the highest-probability amino acid), this strategy preserves the complete probability distribution learned by the model, which is crucial for investigating the mapping from probabilistic parameters to the final amino acid sequence. In this study, we generated a large pool of candidate sequences (N= 5000) via multinomial sampling for each measurement (see Table S4).

1.2 Hyperparameter Sensitivity Analysis for the Loss Weight λ.

In the construction of the composite loss function Loss2 of equation 6 , the hyperparameter λ was selected to balance the contribution of each term. The initial unweighted loss values revealed that Loss1 was approximately sixty times that of Loss2. (e.g., Loss1 $\approx$ 4.58×10^{-3} and Loss2 $\approx$ 7.88×10^{-5}). This disparity caused the optimization gradients to be dominated by Loss1, thereby limiting the model's capacity to learn the critical pairwise coupling information. The primary objective in setting $\lambda =$

65 was to scale Loss2 such that the magnitudes of the two loss terms were approximately equalized. This balancing ensured that the gradients from both loss components had a comparable influence on the model updates.

To justify that the our model are not sensitive to the precise value of λ. we conducted a sensitivity analysis. We evaluated the model's performance for the HLA-DRB5*01:01 allele across four different values of λ: 60, 63, 65, and 66. The performance was assessed using the PCC of the coupling values between the generated L2 sequences and native peptides, alongside an examination of sequence conservation logos and predicted binding affinities. The results presented in Supplementary Table S5 and Supplementary Figure S8, confirm the robustness of our framework. The PCC values and other evaluated metrics remain robust across the tested range of λ, demonstrating that the model's output is not sensitive to the empirical choice of λ.

1.3 MCSA Procedure and Parameter Sensitivity Analysis.

The key parameters governing the MCSA were selected to ensure effective convergence. The initial temperature (T_0 = 15) was calibrated to yield a high initial acceptance probability (~97% based on the Metropolis criterion, $P = \exp(-\Delta E/T)$) for initial objective function values ($\Delta E \approx 0.59$), ensuring sufficient global exploration during the early optimization stages. And this value was further validated through small-scale pilot experiments. A temperature decay factor (α = 0.99) close to 1.0 was chosen to implement a slow annealing strategy. The mutation probability (P_m = 8%) was empirically selected based on robust performance across multiple tests.

To validate the robustness of our design outcomes to these parameter choices, a sensitivity analysis was conducted. We evaluated two alternative parameter sets for the HLA-DRB501:01 allele: Set A (T_0=20, α=0.98, P_m=8%) and Set B (T_0=5, α=0.99, P_m=4%). The binding affinity distributions of peptides generated using these alternative parameters were highly consistent, as shown in Supplementary Figure S5. This consistency confirms that the performance of our design framework is robust to reasonable variations of the MCSA parameters.

1.4 Additional Cross-Validation Using an Alternative Tool (NetMHCIIpan-4.3)

We performed an additional supplementary validation. We utilized another widely recognized prediction tool—NetMHCIIpan-4.3—to re-assess the binding affinities of our designed peptide sequences. We conducted a correlation analysis between the affinity predictions from DeepMHCII and those from NetMHCIIpan-4.3 for specific HLA allotypes (HLA-DRB5*01:01 and HLA-DPA1*01:03_DPB1*02:01). The results, presented in Supplementary Figure S11, show a significant positive correlation with PCC values larger than 0.80 between the predicted affinities from these two distinct tools for our designed peptides. This indicates that, despite using different affinity evaluation models, the binding affinity distributions of our designed sequences and natural MHCII- binding sequences remain similar.

2. Supplementary Results

2.1 Comparison of predicted TM-score

We also conducted an in-depth analysis using multiple confidence metrics from the summary_confidences.json file provided with AlphaFold3 predictions. Among these, the interface predicted TM-score (iPTM) is a key metric, as it reflects both overall structural accuracy and the predicted quality of protein–protein interfaces. For the chain_pair_iptm metric, off-diagonal elements representing the predicted interface quality between the peptide and the two MHCII chains (α and β, respectively). For the chain_iptm metric, which indicates the average predicted confidence of interfaces between the peptide and each MHCII chain.The predicted interface confidence scores (chain_iptm and chain_pair_iptm) for the peptides generated by various design strategies binding to HLA- DRB5*01:0 allele are summarized in Table S6. For comparison, results for the native peptides and random peptides are also included. Notably, design strategies guided by evolutionary information achieved confidence levels comparable to the native peptide, significantly outperforming the random designs.

3. Supplementary Tables

Table S1. Distribution of binding cores across 27 HLA allotypes.

	HLA Allotype	BD2020	$Dset_{pos}$	Dset_bc
1	HLA-DPA1*01:03_DPB1*02:01	0	1066	1066
2	HLA-DPA1*01:03_DPB1*03:01	0	1842	1842
3	HLA-DPA1*01:03_DPB1*04:01	0	3329	3329
4	HLA-DPA1*01:03_DPB1*04:02	0	948	948
5	HLA-DPA1*02:01_DPB1*01:01	0	1760	1760
6	HLA-DPA1*02:01_DPB1*09:01	0	245	245
7	HLA-DPA1*02:01_DPB1*10:01	0	1384	1384
8	HLA-DPA1*02:01_DPB1*14:01	0	2355	2355
9	HLA-DPA1*02:02_DPB1*05:01	0	3269	3269
10	HLA-DQA1*03:01_DQB1*03:02	0	1315	1315
11	HLA-DRB1*01:01	1665	0	1665
12	HLA-DRB1*01:02	0	941	941
13	HLA-DRB1*03:01	913	641	1554
14	HLA-DRB1*04:01	1750	1465	3215
15	HLA-DRB1*04:04	965	0	965
16	HLA-DRB1*04:05	2146	0	2146
17	HLA-DRB1*07:01	1192	507	1699
18	HLA-DRB1*08:01	1194	0	1194
19	HLA-DRB1*08:02	1230	0	1230
20	HLA-DRB1*09:01	1276	0	1276
21	HLA-DRB1*11:01	784	0	784
22	HLA-DRB1*13:02	1408	0	1408
23	HLA-DRB1*15:01	1240	927	2167
24	HLA-DRB3*01:01	579	0	579
25	HLA-DRB3*02:02	278	944	1222
26	HLA-DRB4*01:03	0	985	985
27	HLA-DRB5*01:01	1830	410	2240

Table S2. The conservation values at each position of binding cores designed for the HLA-DRB5*01:01 allele by different strategies.

	Pos1	Pos2	Pos3	Pos4	Pos5	Pos6	Pos7	Pos8	Pos9
Native	1.639	0.277	0.173	0.567	0.205	0.778	0.19	0.158	1.951
FS	1.616	0.0219	0.176	0.564	0.225	0.757	0.174	0.147	1.986
L1	1.623	0.217	0.175	0.505	0.207	0.708	0.18	0.155	1.952
L2	1.62	0.221	0.157	0.548	0.202	0.747	0.181	0.163	1.919
MC1	1.397	0.168	0.142	0.307	0.209	0.553	0.126	0.136	0.775

MC2	1.647	0.206	0.183	0.566	0.214	0.799	0.217	0.223	1.947

Tables S3. PCC values for coupling between designed and native binding cores across HLA allotypes from ten independent training runs.

	HLA Allotype	PCC(Mean ± SD)[a]	
		L1	L2
1	HLA-DPA1*01:03_DPB1*02:01	0.8720±0.0605	0.9580±0.0386
2	HLA-DPA1*01:03_DPB1*03:01	0.7303±0.1553	0.9338±0.1407
3	HLA-DPA1*01:03_DPB1*04:01	0.8273±0.1063	0.8561±0.0885
4	HLA-DPA1*01:03_DPB1*04:02	0.9792±0.0449	0.9942±0.0087
5	HLA-DPA1*02:01_DPB1*01:01	0.9950±0.0062	0.9988±0.0008
6	HLA-DPA1*02:01_DPB1*09:01	0.9951±0.0048	0.9936±0.0079
7	HLA-DPA1*02:01_DPB1*10:01	0.9625±0.0307	0.9529±0.0872
8	HLA-DPA1*02:01_DPB1*14:01	0.9482±0.0216	0.9647±0.0311
9	HLA-DPA1*02:02_DPB1*05:01	0.9812±0.0138	0.9769±0.0448
10	HLA-DQA1*03:01_DQB1*03:02	0.7526±0.1580	0.9233±0.0330
11	HLA-DRB1*01:01	0.9392±0.0539	0.9644±0.0400
12	HLA-DRB1*01:02	0.9674±0.0200	0.9202±0.0443
13	HLA-DRB1*03:01	0.9910±0.0061	0.9785±0.0111
14	HLA-DRB1*04:01	0.8585±0.0844	0.9211±0.0290
15	HLA-DRB1*04:04	0.9176±0.0650	0.9092±0.0488
16	HLA-DRB1*04:05	0.9652±0.0208	0.9332±0.0435
17	HLA-DRB1*07:01	0.8965±0.0305	0.9725±0.0187
18	HLA-DRB1*08:01	0.9895±0.0070	0.9936±0.0045
19	HLA-DRB1*08:02	0.8844±0.0376	0.9652±0.0208
20	HLA-DRB1*09:01	0.8593±0.0532	0.9535±0.0157
21	HLA-DRB1*11:01	0.9760±0.0148	0.9711±0.0237
22	HLA-DRB1*13:02	0.9602±0.0237	0.9888±0.0127
23	HLA-DRB1*15:01	0.8663±0.1617	0.9040±0.0884
24	HLA-DRB3*01:01	0.9992±0.0003	0.8892±0.0932
25	HLA-DRB3*02:02	0.8896±0.0721	0.9753±0.0199
26	HLA-DRB4*01:03	0.8811±0.0934	0.9301±0.1097
27	HLA-DRB5*01:01	0.9861±0.0261	0.9997±0.0002

[a]Data are presented as mean PCC ± standard deviation from ten independent replicates (n=10). *

Tables S4. PCC values for coupling between designed and native binding cores across HLA allotypes , based on five measurements within a single training run.

	HLA Allotype	PCC(Mean ± SD)[a]		
		FS	L1	L2
1	HLA-DPA1*01:03_DPB1*02:01	0.7256±0.0590	0.9304±0.0082	0.9588±0.0079

2	HLA-DPA1*01:03_DPB1*03:01	0.5746±0.2010	0.8523±0.0075	0.9585±0.0071
3	HLA-DPA1*01:03_DPB1*04:01	0.7649±0.0932	0.9421±0.0165	0.8739±0.0264
4	HLA-DPA1*01:03_DPB1*04:02	0.6313±0.2516	0.9894±0.0010	0.9976±0.0005
5	HLA-DPA1*02:01_DPB1*01:01	0.7445±0.1490	0.9983±0.0004	0.9996±0.0001
6	HLA-DPA1*02:01_DPB1*09:01	0.2417±0.2106	0.9975±0.0002	0.9944±0.0001
7	HLA-DPA1*02:01_DPB1*10:01	0.6381±0.1102	0.9886±0.0032	0.9783±0.0024
8	HLA-DPA1*02:01_DPB1*14:01	0.8392±0.0800	0.9449±0.0095	0.9812±0.0018
9	HLA-DPA1*02:02_DPB1*05:01	0.8112±0.0425	0.9911±0.0032	0.9928±0.0007
10	HLA-DQA1*03:01_DQB1*03:02	0.7343±0.2371	0.8570±0.0141	0.8717±0.0158
11	HLA-DRB1*01:01	0.8793±0.0508	0.9746±0.0033	0.9883±0.0023
12	HLA-DRB1*01:02	0.6291±0.1594	0.9807±0.0042	0.9820±0.0062
13	HLA-DRB1*03:01	0.4512±0.1746	0.9973±0.0010	0.9964±0.0012
14	HLA-DRB1*04:01	0.8460±0.0629	0.9216±0.0168	0.9248±0.0128
15	HLA-DRB1*04:04	0.9385±0.0295	0.9156±0.0096	0.9741±0.0041
16	HLA-DRB1*04:05	0.6345±0.1895	0.9732±0.0024	0.9870±0.0020
17	HLA-DRB1*07:01	0.7527±0.0989	0.8891±0.0042	0.8712±0.0121
18	HLA-DRB1*08:01	0.2826±0.0353	0.9930±0.0023	0.9956±0.0013
19	HLA-DRB1*08:02	0.8034±0.0278	0.8523±0.0141	0.9181±0.0130
20	HLA-DRB1*09:01	0.8542±0.0732	0.8990±0.0208	0.9392±0.0072
21	HLA-DRB1*11:01	0.8993±0.0565	0.9788±0.0033	0.9922±0.0008
22	HLA-DRB1*13:02	0.7928±0.0628	0.9556±0.0169	0.9786±0.0037
23	HLA-DRB1*15:01	0.9359±0.0601	0.9892±0.0036	0.9913±0.0011
24	HLA-DRB3*01:01	0.9077±0.0868	0.9997±0.0000	0.9997±0.0000
25	HLA-DRB3*02:02	0.8051±0.0266	0.7609±0.0124	0.9578±0.0096
26	HLA-DRB4*01:03	0.6384±0.2490	0.9897±0.0034	0.9885±0.0024
27	HLA-DRB5*01:01	0.6393±0.2070	0.9974±0.0017	0.9971±0.0006

[a]Data are presented as mean PCC ± standard deviation from five independent replicates (n=5) within a single training run. *

Table S5. Evaluation metrics for L2-designed peptides across λ values for HLA-DRB5*01:01. Metrics include the coupling Pearson correlation coefficient (PCC) to natural sequences, and the central tendency (mean and median) of the predicted binding affinity.

	PCC	Affinity Score (mean)	Affinity Score (median)
λ = 60	0.998	0.446	0.457
λ = 63	0.997	0.452	0.465
λ = 65	0.997	0.452	0.464
λ = 66	0.998	0.446	0.458

Table S6. Mean values of chain_iptm, chain_pair_iptm of HLA-DRB5*01:01-binding cores complex.

	chain_iptm	chain pair iptm (α chain)	chain pair iptm (β chain)

Native	0.89	0.89	0.91
FS	0.89	0.89	0.90
L1	0.91	0.90	0.91
L2	0.88	0.88	0.88
Random	0.56	0.52	0.53
MC1	0.58	0.58	0.60
MC2	0.90	0.90	0.91

4. Supplementary Figures

A B

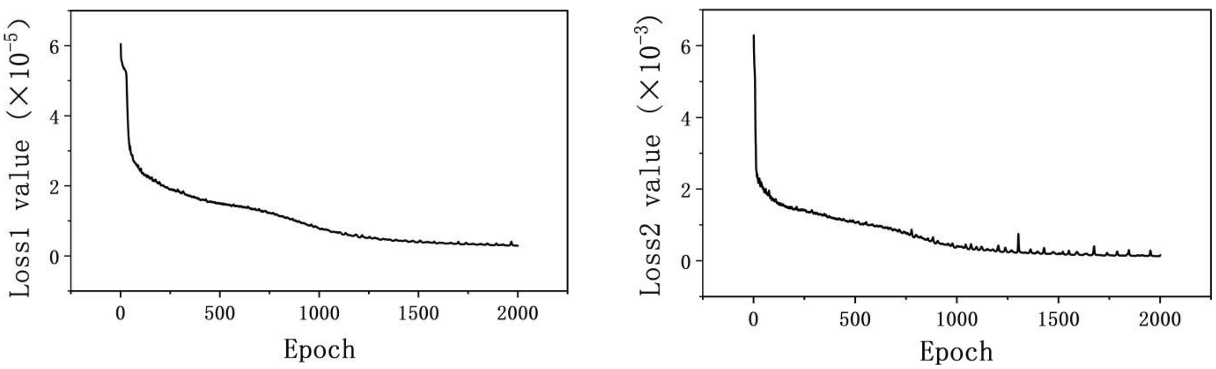

Figure S1. The loss function of the neural network changes with the number of training epochs. (A) Loss change in amino acid frequency. (B) Loss change in joint frequency of residue pairs.

A B

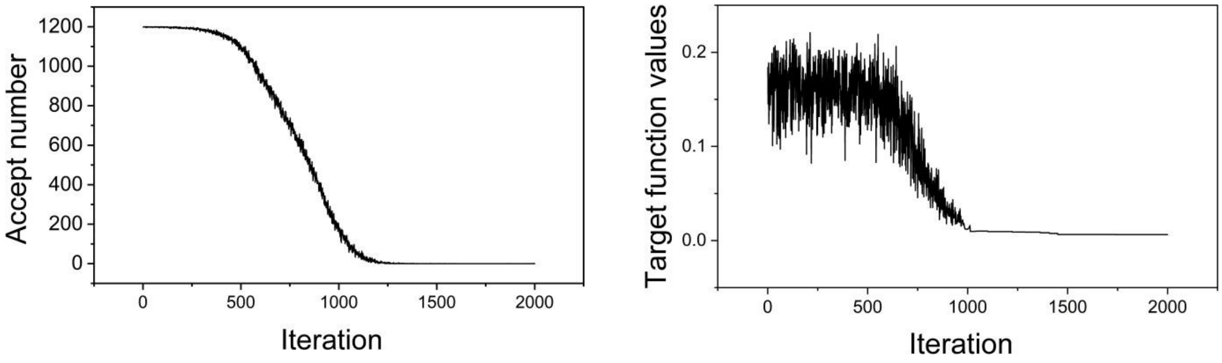

Figure S2. Convergence of acceptance number and objective function values in Monte Carlo simulated annealing optimization. (A) The acceptance number of new solutions decreases progressively with increasing iteration count until convergence is reached. (B) The objective function exhibits significant fluctuations, reflecting the exploration of diverse solutions at higher temperatures. As iterations progress, these fluctuations diminish, and the objective function converges to a stable value.

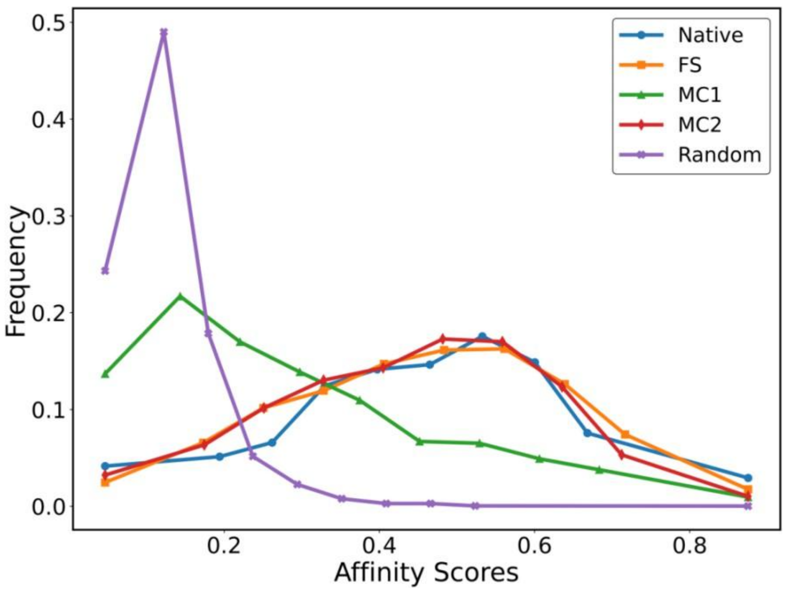

Figure S3. Binding affinity distributions of designed sequences, native sequences, and random sequences for the HLA-DRB5*01:01.

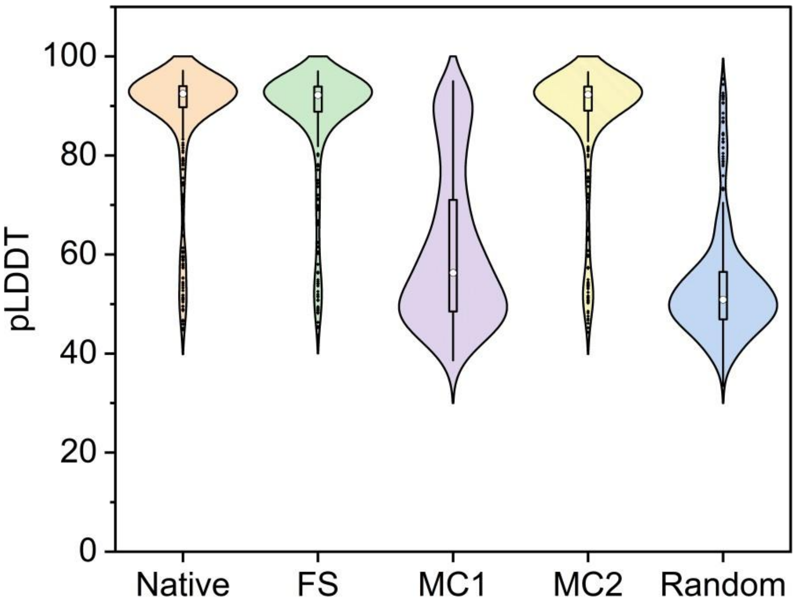

Figure S4. The distribution of pLDDT scores for five peptide groups binding to the HLA-DRB5*01:01 allele, with 200 sequences sampled from each group. For MHCII-peptide complexes, the results show that the average pLDDT score of MC2 sequences is higher than that of MC1 sequences, and the pLDDT score of MC1 sequences is higher than that of random sequences.

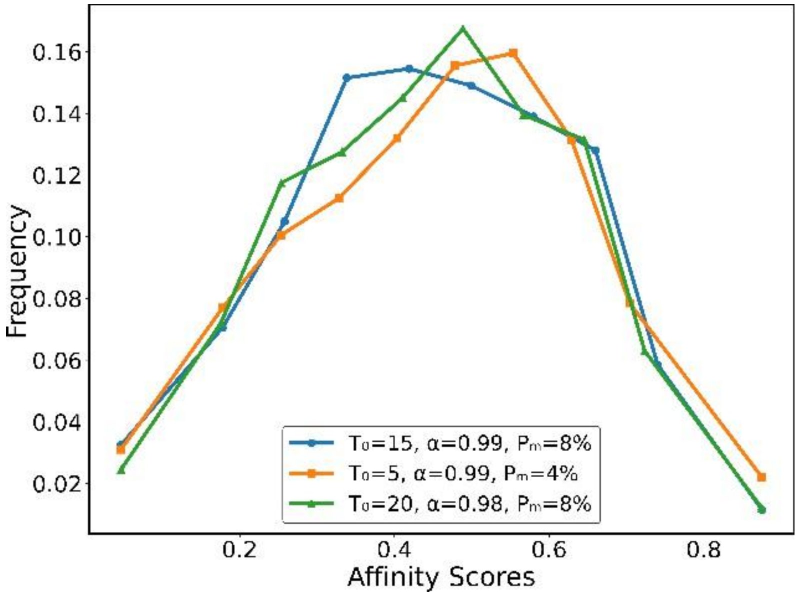

Figure S5. Affinity distributions of peptides designed for HLA-DRB5*01:01 using different MCSA parameters.

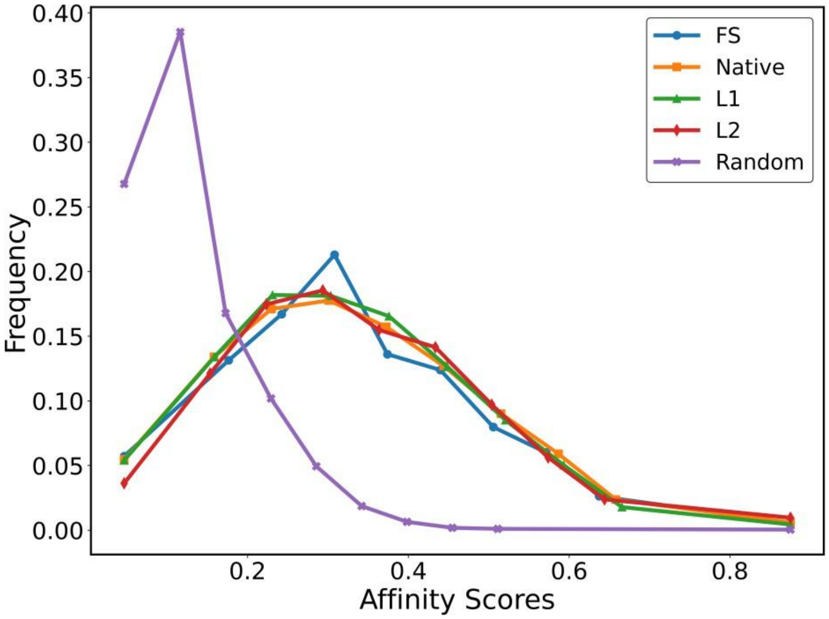

Figure S6. Binding affinity distributions of the designed sequences, and native sequences and random sequences for HLA-DPA1*01:03_DPB1*02:01.

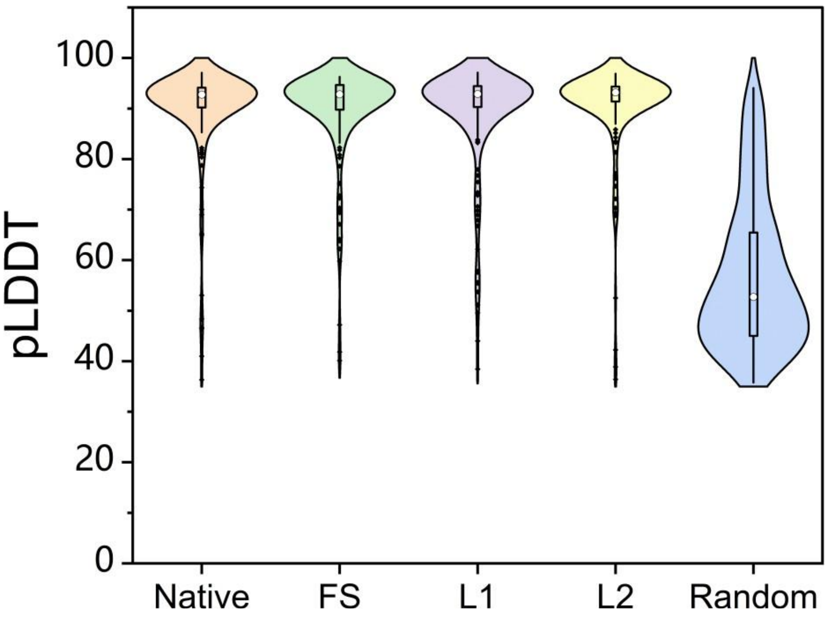

Figure S7. The distribution of pLDDT scores for five peptide groups binding to HLA-DPA1*01:03_DPB1*02:01, with 200 sequences sampled from each group. For MHCII-peptide complexes, the results show that the average pLDDT scores of binding cores designed by different strategies are >90.

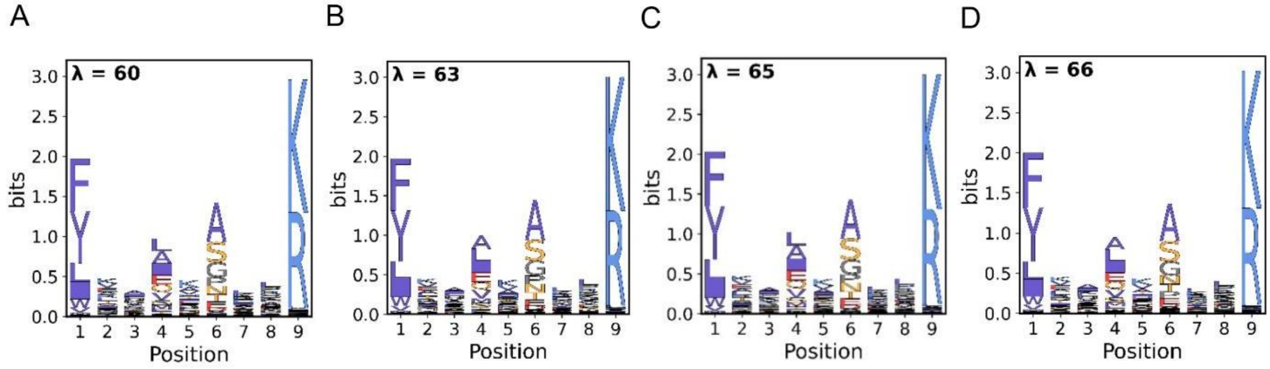

Figure S8. First-order conservation profiles of 9-mer binding cores which designed by the different loss weight for HLA-DRB5*01:01 allele. (A) λ = 60. (B) λ = 63. (C) λ = 65. (D) λ = 66.

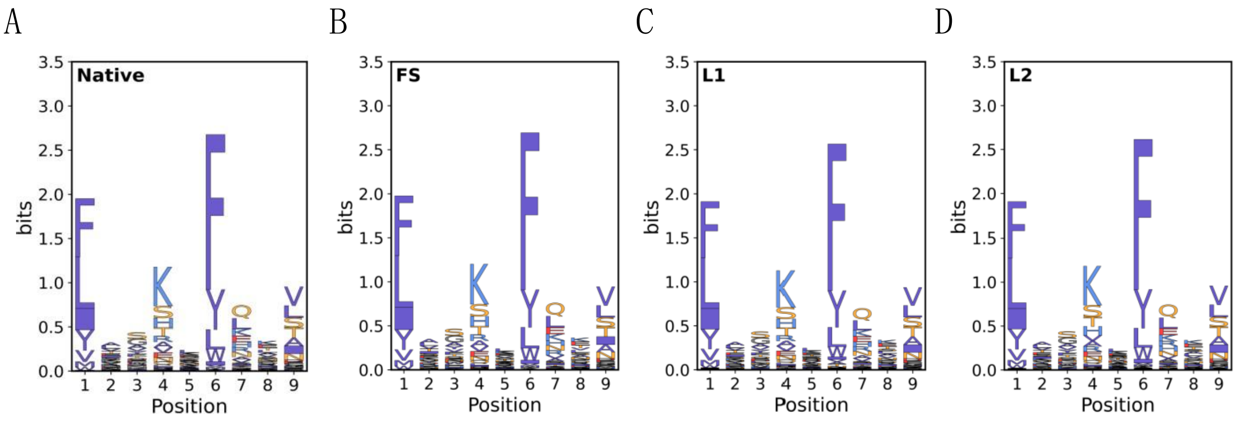

Figure S9. First-order conservation profile of 9-mer binding cores with different strategies for HLA-DPA1*01:03_DPB1*02:01. (A) Native sequences. (B) FS sequences. (C) L1 sequences. (D) L2 sequences.

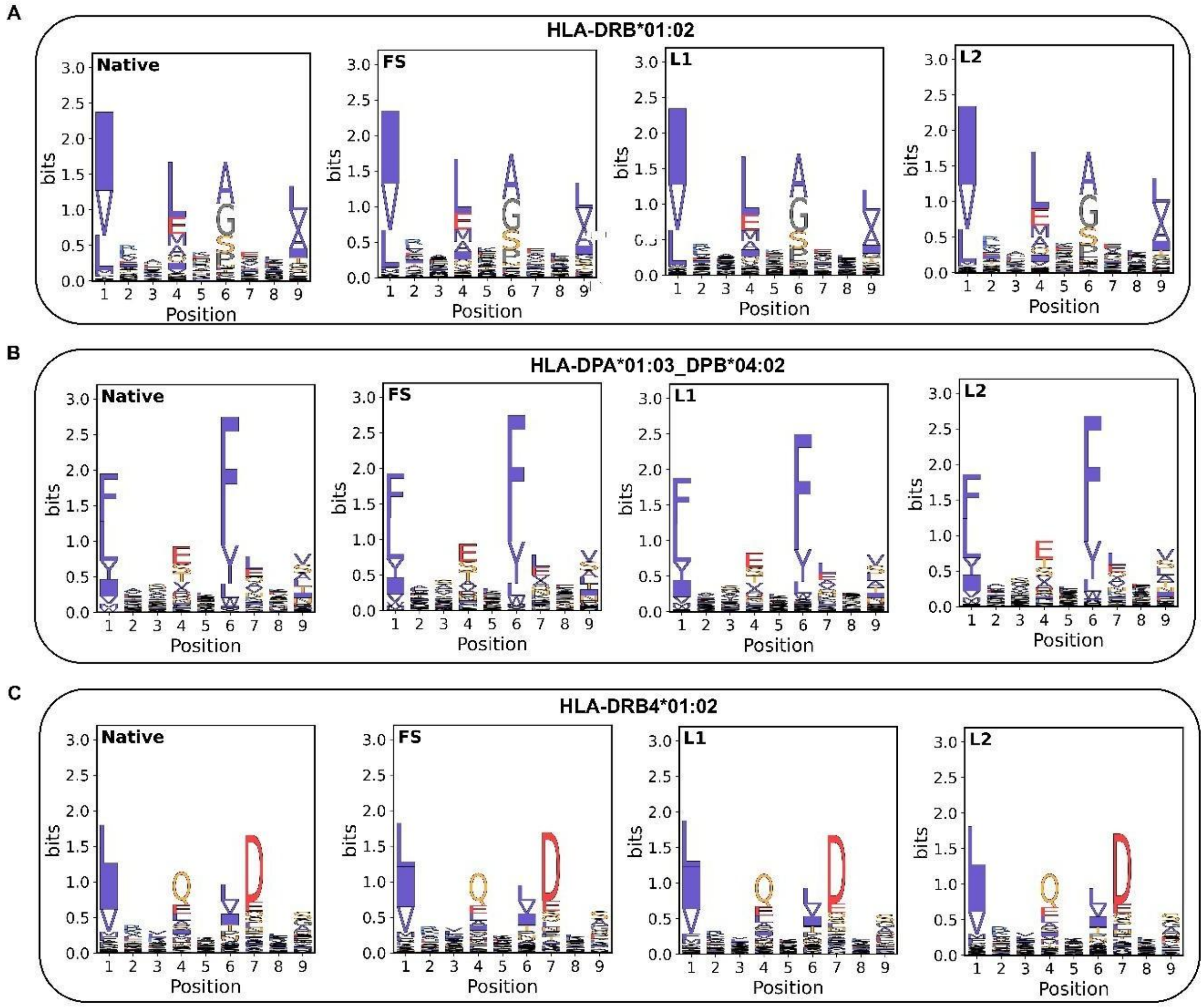

Figure S10. First-order conservation profiles of 9-mer binding cores with different strategies for (A) HLA-DRB*01:02, (B) HLA-DPA1*01:03_DPB1*04:02 and (C) HLA-DRB4*01:02.

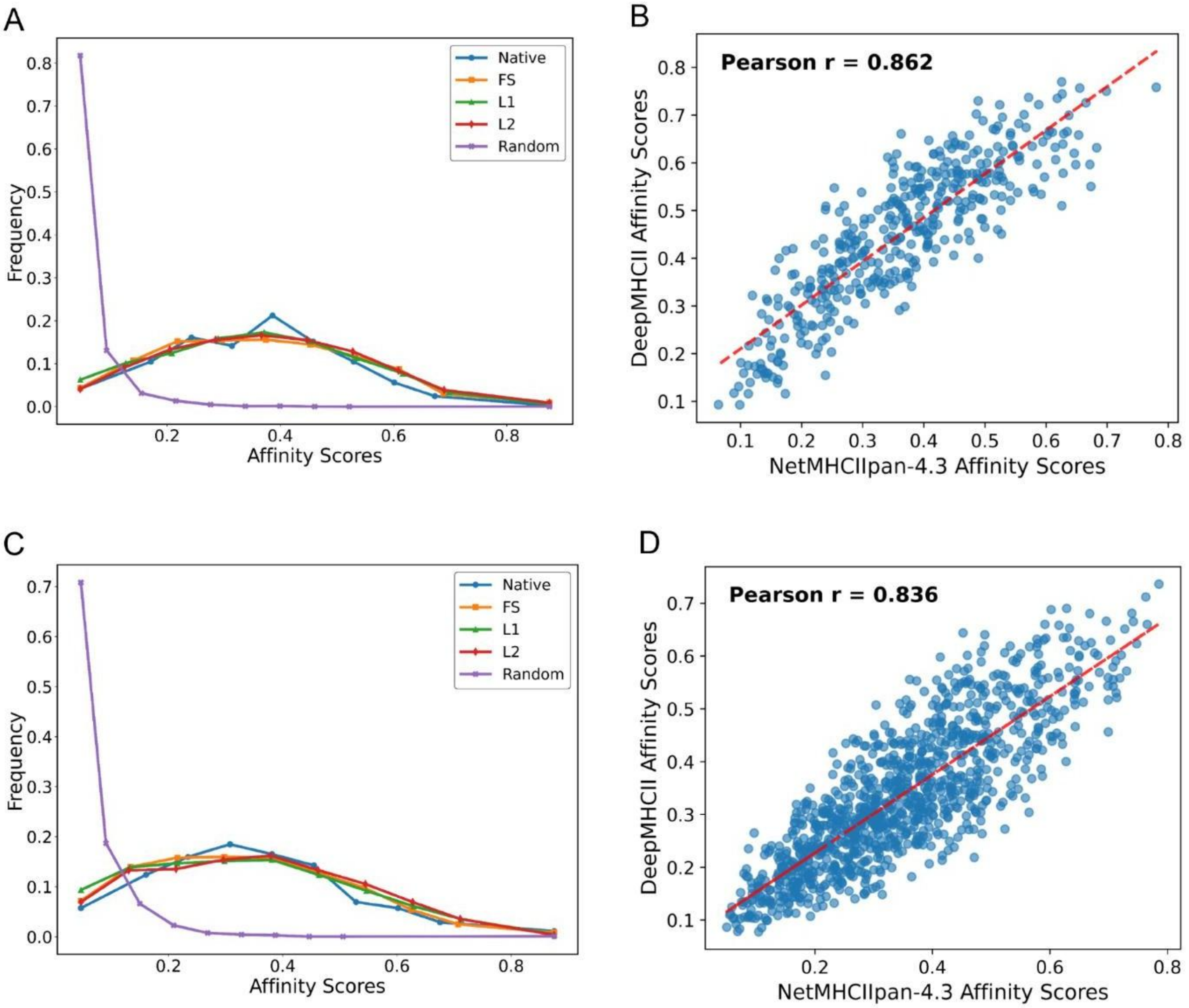

Figure S11. Validation of peptide-MHCII binding predictions using NetMHCIIpan-4.3. (A) Predicted distribution of binding affinities for HLA-DRB5*01:01. (B) Correlation of affinity predictions between DeepMHCII and NetMHCIIpan-4.3 (Pearson r =0.862). (C) Predicted distribution of binding affinities for DPA1*01:03_DPB1*02:01. (D) Correlation of affinity predictions between DeepMHCII and NetMHCIIpan-4.3 (Pearson r=0.836).

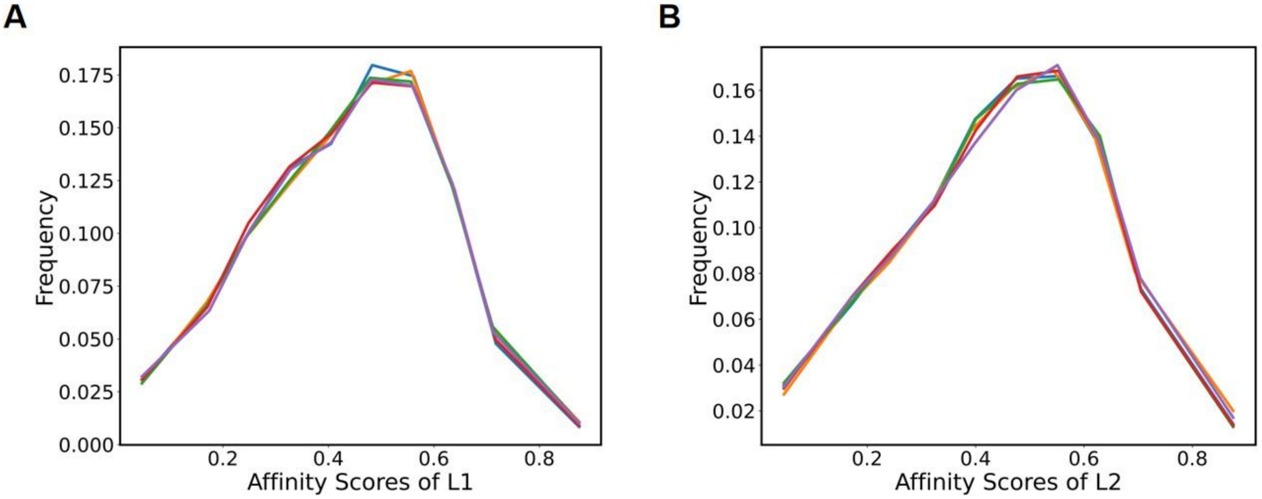

Figure S12. The affinity frequency distributions of five independently generated sets of L1 sequences and five sets of L2 sequences with the HLA-DRB5*01:01 allele within a train. (A) The affinity frequency distributions of all five L1 sequence sets with the HLA-DRB5*01:01 allele show central values around 0.45. (B) The affinity frequency distributions of all five L2 sequence sets with the HLA-DRB5*01:01 allele show central values around 0.5.

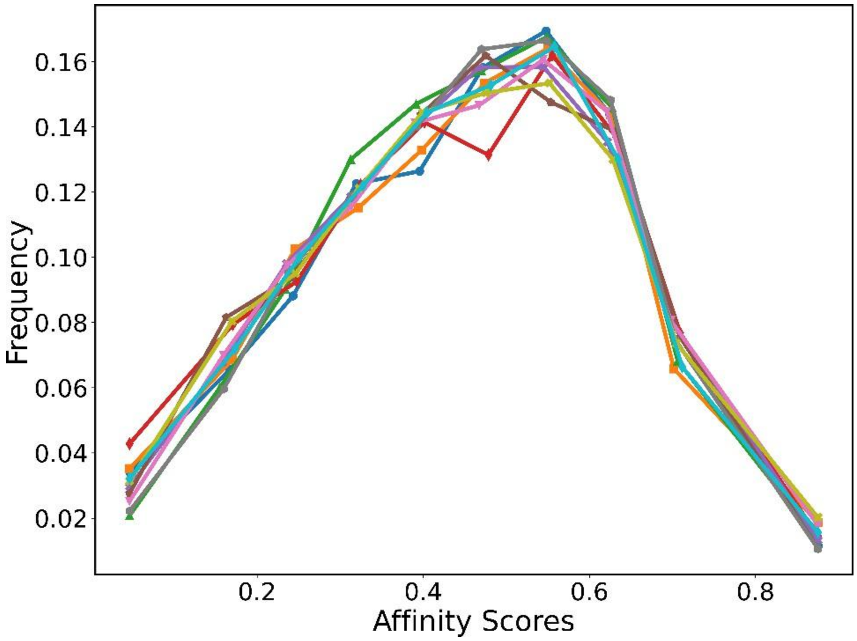

Figure S13. The affinity frequency distributions of generated L2 sequences for the HLA-DRB5*01:01 allele from ten independently trains.

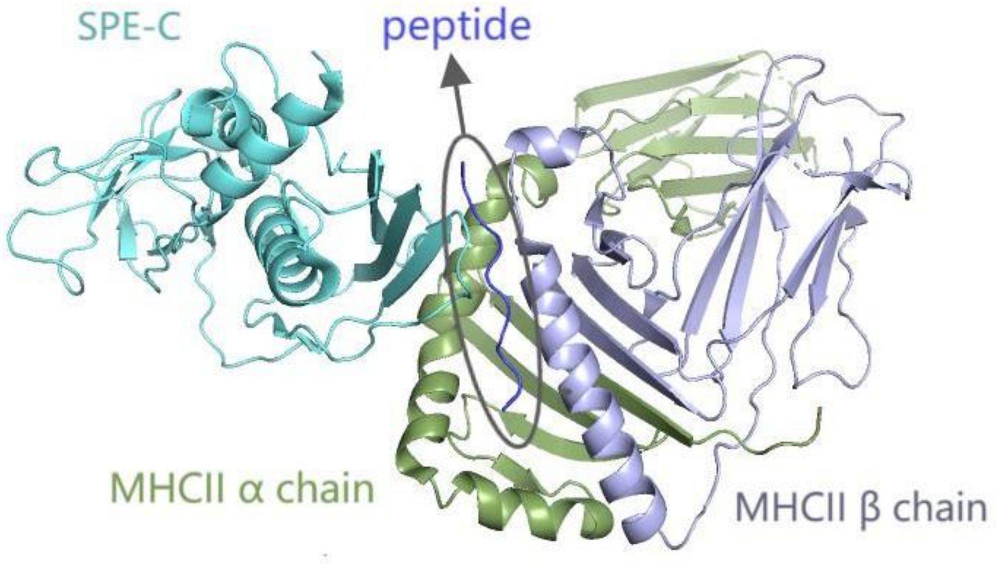

Figure S14. The predicted structure of MHCII-Peptide-SPE-C ternary complex. The predicted complex structure of designed peptide (IKLLASLRK) binding with MHCII and SPE-C retains a structural architecture highly similar to the native complex (PDB ID: 1HQR).

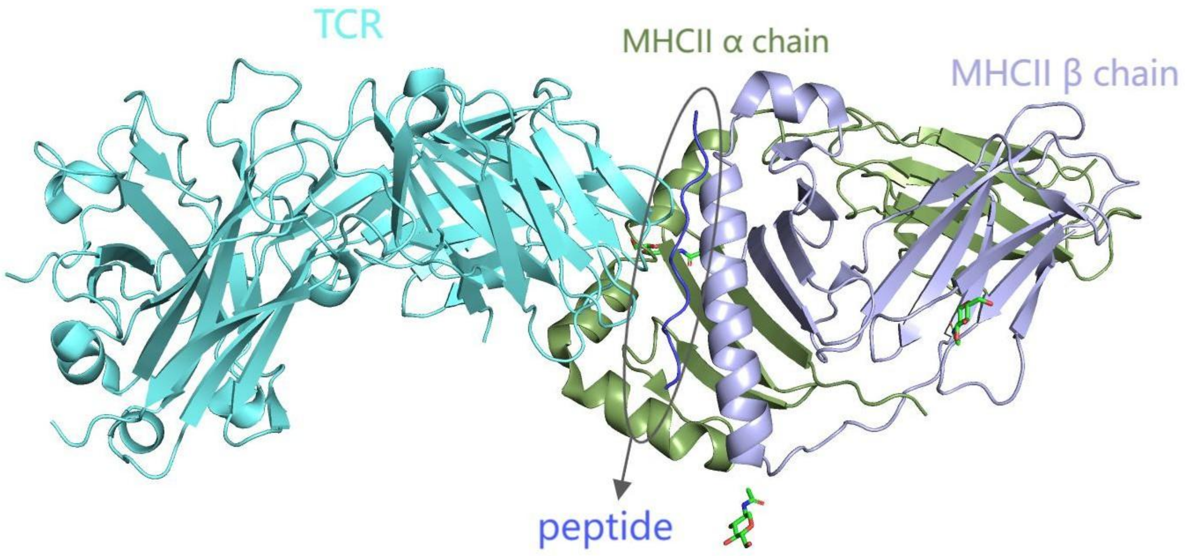

Figure S15. The predicted structure of the MHCII-peptide-TCR ternary complex. The predicted complex structure of designed peptide (FTYNQFMKV) binding with MHCII and TCR retains a structural architecture highly similar to the native complex (PDB ID: 4P4K).

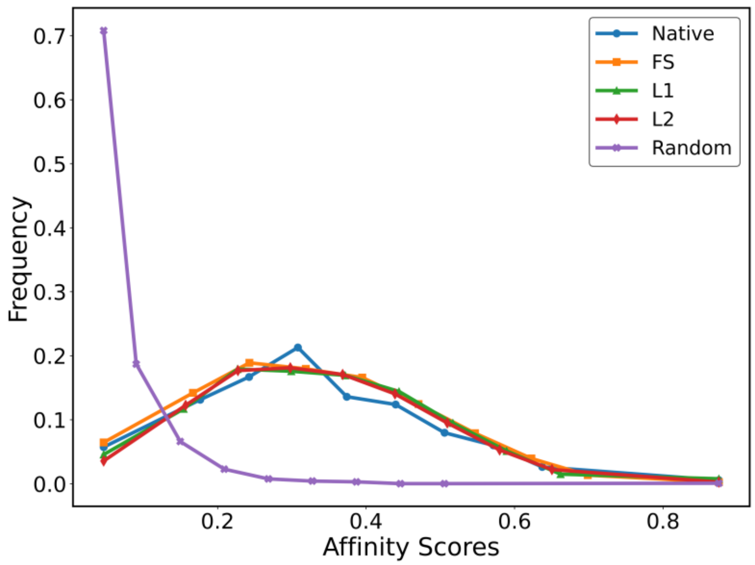

Figure S16. Predicted distribution of binding affinities for DPA1*01:03_DPB1*02:01 by excluding overlapping data with training data of DeepMHCII .
